# Lysozyme Photochemistry as a Function of Temperature. The Protective Effect of Nanoparticles on Lysozyme Photostability

**DOI:** 10.1371/journal.pone.0144454

**Published:** 2015-12-14

**Authors:** Catarina Oliveira Silva, Steffen B. Petersen, Catarina Pinto Reis, Patrícia Rijo, Jesús Molpeceres, Henrik Vorum, Maria Teresa Neves-Petersen

**Affiliations:** 1 Research Center for Biosciences & Health Technologies, Universidade Lusófona, Lisboa, 1749–024, Portugal; 2 Department of Biomedical Sciences, Faculty of Pharmacy, University of Alcalá, 28871 Alcalá de Henares, Spain; 3 Medical Photonics Lab, Department of Health Science and Technology, Faculty of Medicine, Aalborg University, Fredrik Bajers vej 7, DK-9220, Aalborg, Denmark; 4 IBEB, Biophysics and Biomedical Engineering, Faculty of Sciences, University of Lisbon, 1749–016, Lisbon, Portugal; 5 Department of Ophthalmology, Aalborg University, Hobrovej 18–22, 9000 Aalborg, Denmark; 6 Department of Clinical Medicine, Aalborg University, Søndre Skovvej 15, 9000 Aalborg, Denmark; Islamic Azad University-Mashhad Branch, Mashhad, Iran, ISLAMIC REPUBLIC OF IRAN

## Abstract

The presence of aromatic residues and their close spatial proximity to disulphide bridges makes hen egg white lysozyme labile to UV excitation. UVB induced photo-oxidation of tryptophan and tyrosine residues leads to photochemical products, such as, kynurenine, N–formylkynurenine and dityrosine and to the disruption of disulphide bridges in proteins. We here report that lysozyme UV induced photochemistry is modulated by temperature, excitation power, illumination time, excitation wavelength and by the presence of plasmonic quencher surfaces, such as gold, and by the presence of natural fluorescence quenchers, such as hyaluronic acid and oleic acid. We show evidence that the photo-oxidation effects triggered by 295 nm at 20°C are reversible and non-reversible at 10°C, 25°C and 30°C. This paper provides evidence that the 295 nm damage threshold of lysozyme lies between 0.1 μW and 0.3 μW. Protein conformational changes induced by temperature and UV light have been detected upon monitoring changes in the fluorescence emission spectra of lysozyme tryptophan residues and SYPRO^®^ Orange. Lysozyme has been conjugated onto gold nanoparticles, coated with hyaluronic acid and oleic acid (HAOA). Steady state and time resolved fluorescence studies of free and conjugated lysozyme onto HAOA gold nanoparticles reveals that the presence of the polymer decreased the rate of the observed photochemical reactions and induced a preference for short fluorescence decay lifetimes. Size and surface charge of the HAOA gold nanoparticles have been determined by dynamic light scattering and zeta potential measurements. TEM analysis of the particles confirms the presence of a gold core surrounded by a HAOA matrix. We conclude that HAOA gold nanoparticles may efficiently protect lysozyme from the photochemical effects of UVB light and this nanocarrier could be potentially applied to other proteins with clinical relevance. In addition, this study confirms that the temperature plays a critical role in the photochemical pathways a protein enters upon UV excitation.

## Introduction

The fluorescence of aromatic amino acids in proteins can be used to monitor protein conformational changes, to determine the protein’s melting temperature, to detect solvent accessibility changes and to unravel the onset of photochemical pathways. Their fluorescence spectral properties can be modulated by solvent polarity (tryptophan in particular) and by the presence of fluorescence quenchers. Spectral shifts are monitored in order to probe, e.g., for protein-receptor binding, protein-protein dimerization and protein-metal binding [1]. Extrinsic fluorescence probes such as SYPRO^®^ Orange and 8-Anilinonaphthalene-1-sulfonic acid (ANS) can also reveal protein conformation changes induced by, e.g., ligand binding, temperature, pH and UV light, as their fluorescent emission is enhanced upon binding to hydrophobic regions of the protein [2]. On the other hand, water strongly quenches their fluorescence.

UVB induced photo-oxidation of tryptophan (Trp) and tyrosine (Tyr) residues leads to the formation of photochemical products, such as, kynurenine (Kyn), N—formylkynurenine (NFK), singlet oxygen, 3α-hydroperoxypyrroloindole, 3α-dihydroxypyrroloindole, hydroxyl radicals and dityrosine (DT) [3, 4]. The presence and the kinetics of formation of NFK, Kyn and DT can be monitored by fluorescence spectroscopy (Table 1). Furthermore, UVB induced photo-oxidation of Trp, Tyr and Phenylalanine (Phe) residues leads to electron ejection from their side chains [5]. Such electron can be captured by disulphide (SS) bridges, leading to a transient disulphide electron adduct, and ultimately to the reduction of the SS bridges [5]. Since SS bridges are one of the best quenchers of protein fluorescence, this can lead to a fluorescence emission intensity increase [5, 6, 7]. On the other hand, the conversion of Trp and Tyr into their photoproducts leads to a decrease of the original fluorescence emission intensity [8]. Different photochemical pathways will lead to an increase or a decrease of the protein’s fluorescence emission intensity. The triggered pathways will depend on, e.g., the excitation wavelength, the irradiance level (power per unit area) and the temperature [9], leading lead to reversible [8] or irreversible changes [10].

**Table 1:** Absorption and fluorescence spectral characteristics of N-formylkynurenine (NFK), dityrosine (DT) and kynurenine (Kyn), according to the literature [25–27].

Photo degradation product	Absorption (nm)	Fluorescence emission (nm)
NFK: N-formylkynurenine	261, 322	400–440
DT: Dityrosine	284, 316	400–409
Kyn: Kynurenine	258, 360	434–480

**Fig 1:**
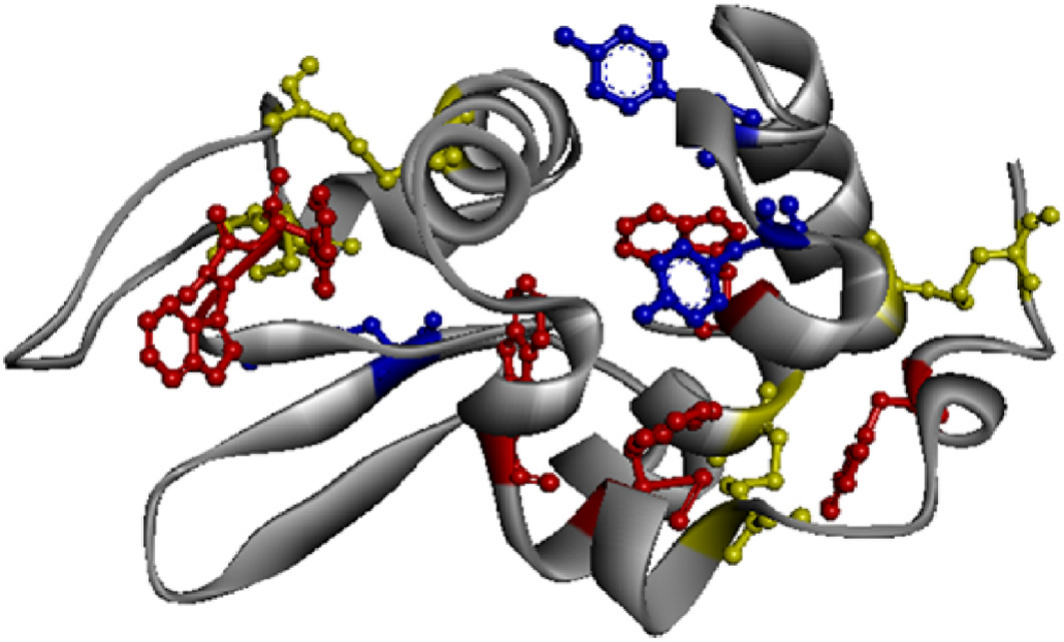
LYZ molecular structure, according to (2LYZ.pdb). Aromatic residues are represented by different colors: Trp (red), Tyr (blue), Cys (yellow).

Hen egg white lysozyme (LYZ) is a small size (129 amino acids) monomeric catalytic enzyme displaying 4 SS, 6 Trp and 3 Tyr residues (see Fig 1). LYZ structural changes induced by temperature, UVB light, pH and ionic strength haves been previously reported [2, 9–12]. LYZ is described to show a two-stage denaturation induced by temperature and a melting temperature ranging from 52°C to 77°C, depending on pH [10, 13]. The studies done by Neves-Petersen et al. confirmed that UVB excitation of aromatic residues leads to the disruption of SS bridges. Such disruption leads to changes in the proteins fluorescence emission intensity and to the formation of photo products of Trp and Tyr residues [5, 6, 8, 14, 15].

There is an interest in protecting proteins from photo damage, since some of these proteins might be used as biosensors, drugs or simply as enzymes. Protein photochemistry can be modulated by the presence of plasmonic surfaces such as gold [16, 17] and by the presence of fluorescence quenchers such as hyaluronic acid (HA, [18]) and oleic acid (OA, [19]). HA is reported to be a very good fluorescence quencher, as well as to confer structural stability to proteins [20]. LYZ has a high isoelectric point (pI) value around 11.1 [21]. This allows for LYZ binding onto the surface of negative charged multifunctional gold nanoparticles via electrostatic attractive interactions (at pH < pI), putatively protecting it from UVB induced photochemistry.

In this study we have monitored the time dependent effect of continuous 295 nm excitation of LYZ on the protein’s fluorescence emission intensity, as a function of irradiance level, temperature and excitation wavelength. The kinetics of such processes are analysed and compared. The reversibility of the light induced pathways is observed to be temperature dependent in the chosen temperature range from 10–30°C. Protein conformational changes induced by 295 nm and temperature have been monitored using Trp as an intrinsic molecular probe and SYPRO^®^ Orange as an extrinsic molecular probe. The formation of photoproducts such as NKF and Kyn has been monitored as a function of excitation time. Furthermore, LYZ has been coupled to gold nanoparticles coated with HA and OA. The putative protective effect of the coated gold nanoparticles against photochemistry is investigated. The kinetics and extent of light induced processes and the protein fluorescence lifetimes alone and when coupled to the gold nanoparticles are compared.

## Materials and Methods

### Materials

Gold (III) chloride trihydrate (HAuCl_4_) (PubChem ID: 24895143; Product number: G4022), sodium citrate dihydrate (C_6_H_5_Na_3_O_7_) (PubChem ID: 24901436; Product number: W302600), L-ascorbic acid (L-AA) (PubChem ID: 24891246; Product number: A7506), silver nitrate (AgNO_3_) (PubChem ID: 24852543; Product number: S0139), hyaluronic acid (HA) sodium salt from *Streptococcus equi* (MW: 7,000–250,000 g.mol^-1^) (PubChem ID: 24878223; Product number: 53747), oleic acid (OA) (MW: 282.46 g.mol^-1^) (PubChem ID: 24886786; Product number: 75090), Lysozyme from hen egg white, in powder form, (LYZ, MW: 14.3 kDa) (Enzyme number:3.2.1.17; Product number: L6876) were all supplied by Sigma-Aldrich (Steinheim, Germany). SYPRO^®^ Orange Protein Gel Stain (5,000X Concentrate in DMSO) was purchased from Life Technologies as the molecular probe for protein conformational studies. The water used for buffer preparation was purified through a Millipore system.

### Preparation of LYZ stock solution and LYZ-conjugated gold nanoparticles

A 10 μM (0.15 mg/mL) stock solution of LYZ was prepared in 2 mM Phosphate Buffer Saline (PBS) at pH 7.4. After mixing directly the protein with the buffer, the solution had pH 6.0. In order to prepare LYZ-conjugated gold nanoparticles, the stock solution of the protein at 10 μM was mixed with the gold nanoparticles solution (0.22 mM) and hyaluronic acid-oleic acid (HAOA) polymer solution at a 1:1:1 (v/v/v, concentration of 1 mg/mL, for each polymer) ratio and allowed to interact for 30 min at room temperature. Gold nanoparticles were prepared using produced by seed-growth method, described elsewhere [22], with some modifications (paper in preparation). The solution was centrifuged twice at 500 g for 20 min in a FV2400 Microspin (BioSan, Riga, Latvia) to remove unbound peptides. The pellet was re-suspended in PBS buffer (pH 7.4). LYZ stock solution was stored at 4–8°C until further use.

### LYZ structure analysis and gold nanoparticles structure design

The crystallographic data used for the display of the 3D protein structure (Fig 1) was extracted from (2LYZ.pdb) (structure of hen egg-white lysozyme, [23]) using Discovery Studio 4.1 (Accelrys Software, San Diego, CA, USA). Distances between protein residues were obtained by using the monitor tool in the program to determine the distance between atoms in the 3D structure (see Table 2). As for LYZ-conjugated HAOA gold nanoparticles structural design, Adobe Illustrator CS5 (Adobe Systems Software Ireland Ltd.) was used as the graphic design software.

**Table 2:** Shortest spatial distances between disulphide bonds and aromatic residues (tryptophan and tyrosine) in LYZ (2LYZ.pdb). The shortest distances (< 12 Å) between atoms of each pair of elements (Trp, Tyr and disulphide bonds) were considered. For Trp and Tyr residues, only one of the atoms belonging to the indole and benzene rings were considered, and for SS bonds one of SG atoms. (W = Trp; Y = Tyr).

Disulphide Bond	Aromatic Residue	Distance (Å)
**C30-C115**	W123 (CD1)	3.3
	W111(CD1)	5.6
	W23 (CD1)	10.7
	W28 (CD2)	10.2
**C6- C127**	W123 (CD2)	9.2
**C76-C94**	W108(CH2)	8.0
	Y20 (CD2)	11.9
	W63 (CH2)	6.6
**C64-C80**	Y53 (CD2)	4.1
	W62 (CD2)	10.7
	W63 (CD2)	7.7

### Steady-state fluorescence spectroscopy studies

Steady-state fluorescence emission spectra were collected upon excitation of the aromatic pool of the protein at 295 nm. Excitation spectra, with a fixed wavelength at 330 nm (or 350 nm, for thermal ramp), were also monitored. All measurements were conducted on a Felix fluorescence RTC 2000 spectrometer (Photon Technology International, Canada, Inc.347 Consortium Court London, Ontario N6E 2S8) with a T-configuration, using a 75-W Xenon arc lamp coupled to a monochromator. The samples were analyzed in a cuvette of 1 cm light path and were magnetically stirred at 200 rpm in order to secure homogeneous excitation. All slits were set to 5 nm.

#### Thermal unfolding studies and melting point of LYZ

**Fig 2:**
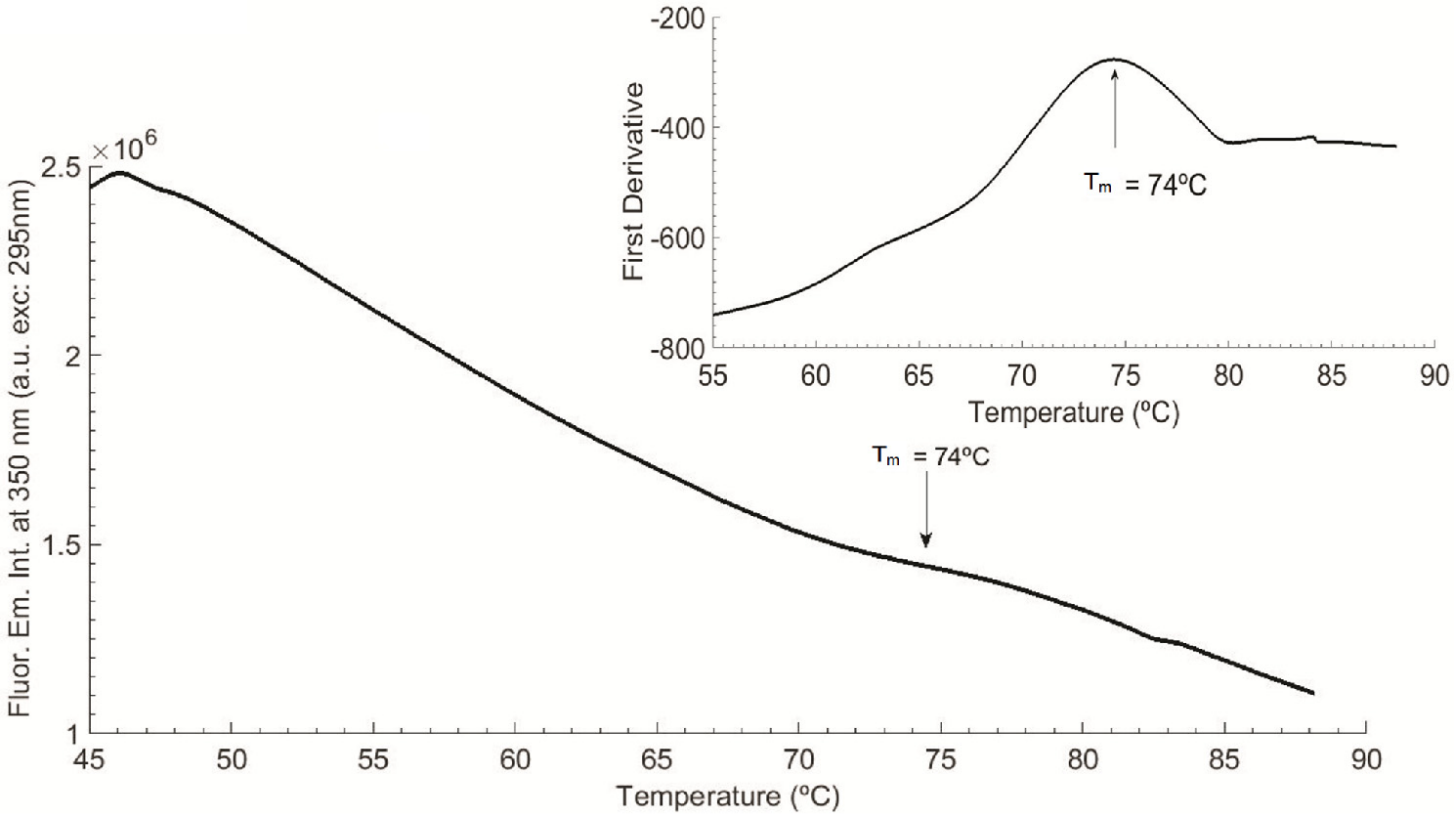
LYZ thermal ramp and data first derivative from 45–90°C (T_m_ = 74°C), with heating rate fixed at 1°C/min. Fluorescence excitation was fixed at 295 nm and fluorescence emission at 350 nm and excitation slit was set at 0.1 mm (0.1 μW).

**Fig 3:**
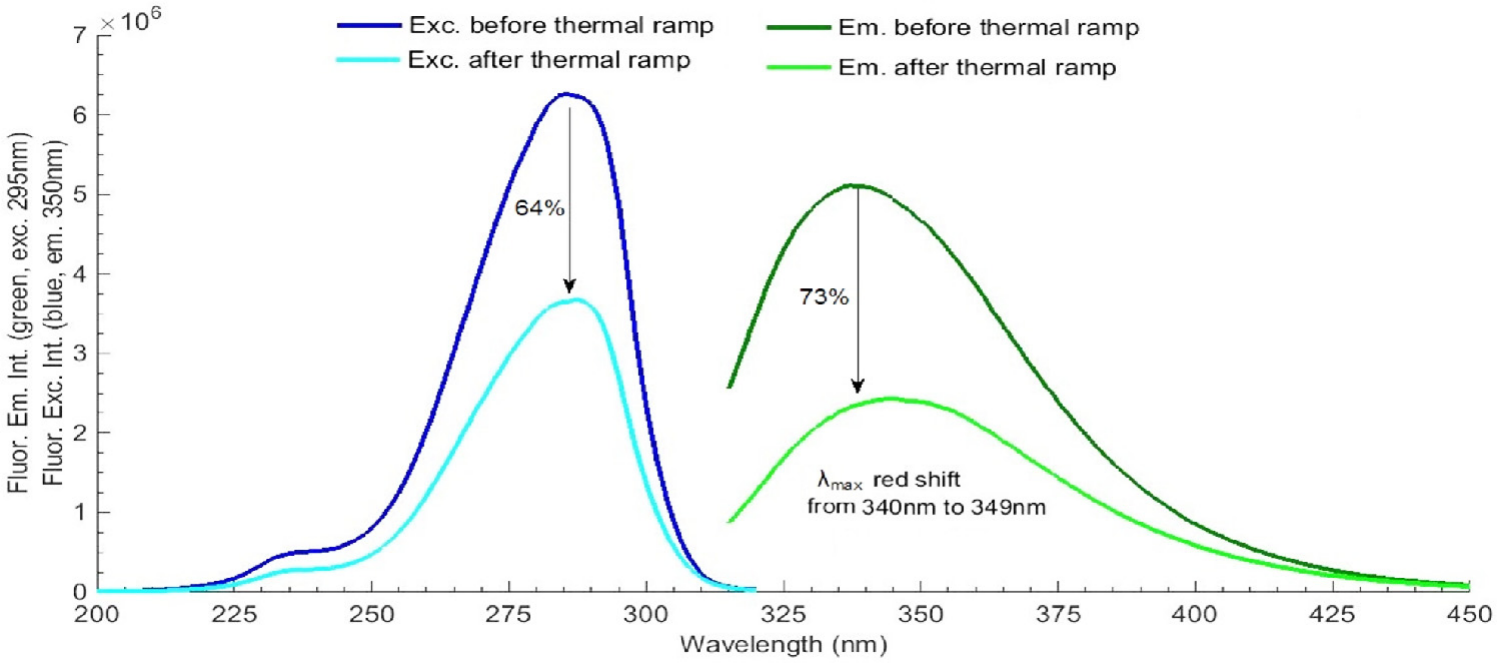
LYZ fluorescence excitation and emission spectra, before and after the thermal ramp at 45–90°C. Fluorescence excitation was fixed at 295 nm and fluorescence emission at 350 nm and excitation slit was set at 0.1 mm (0.1 μW).

LYZ thermal unfolding studies were conducted in order to determine the melting point of the protein prior to illumination (see Fig 2). The fluorescence emission intensity at 350 nm (exc. 295 nm) of fresh LYZ sample (non-illuminated) was monitor from 45°C to 90°C. The heating rate was fixed at 1°C/min. Excitation slit size was set at 0.1 mm (equivalent lamp power of 0.1 μW). Trp emission is usually used as a probe for protein conformational changes and can be used to determine the melting temperature of the protein. First derivative was conducted in Matlab version R2014b (MathWorks, Massachusetts, USA) for calculation of the melting temperature, according to the mid-point value in°C. Fluorescence excitation (em. fixed at 350 nm) and emission (exc. fixed at 295 nm) spectra of LYZ were analysed before and after thermal unfolding and changes in fluorescence intensity were quantified (see Fig 3).

#### Continuous 295 nm illumination of LYZ

**Fig 4:**
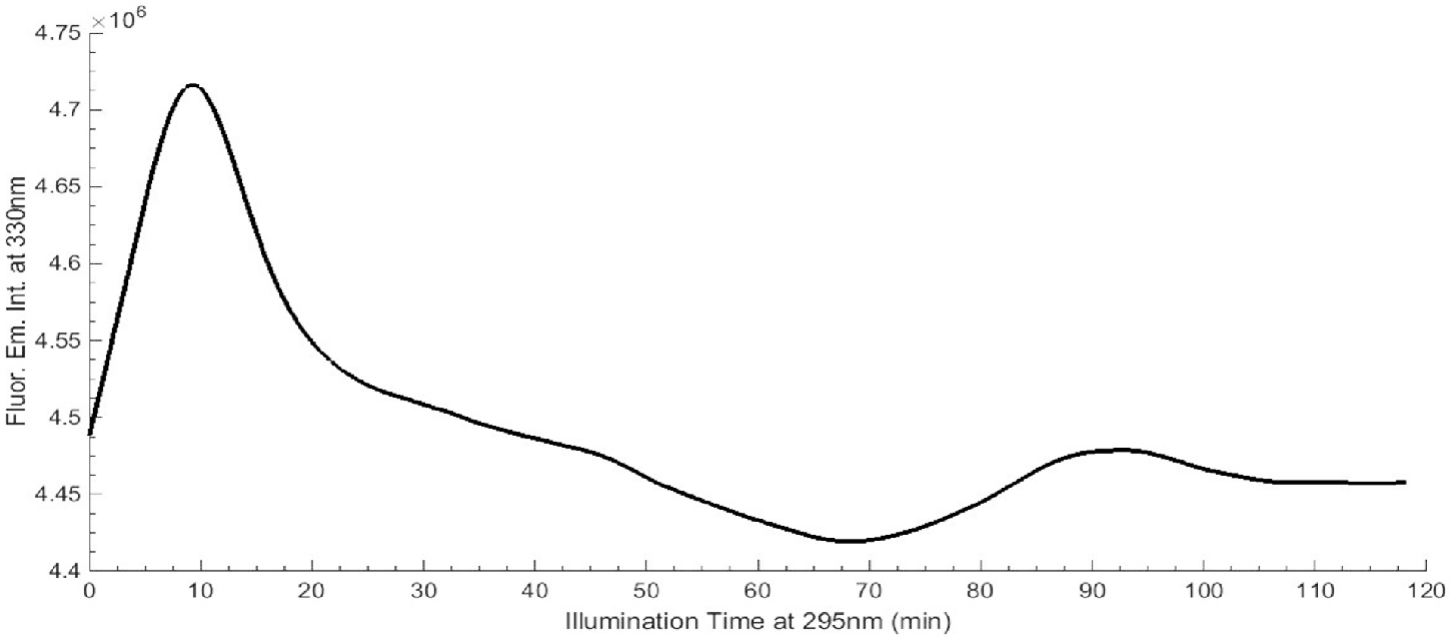
Continuous 295 nm excitation of LYZ at 20°C, for 2 hours. Fluorescence emission at 330 nm and excitation slit was set at 0.1 mm (0.1 μW).

**Fig 5:**
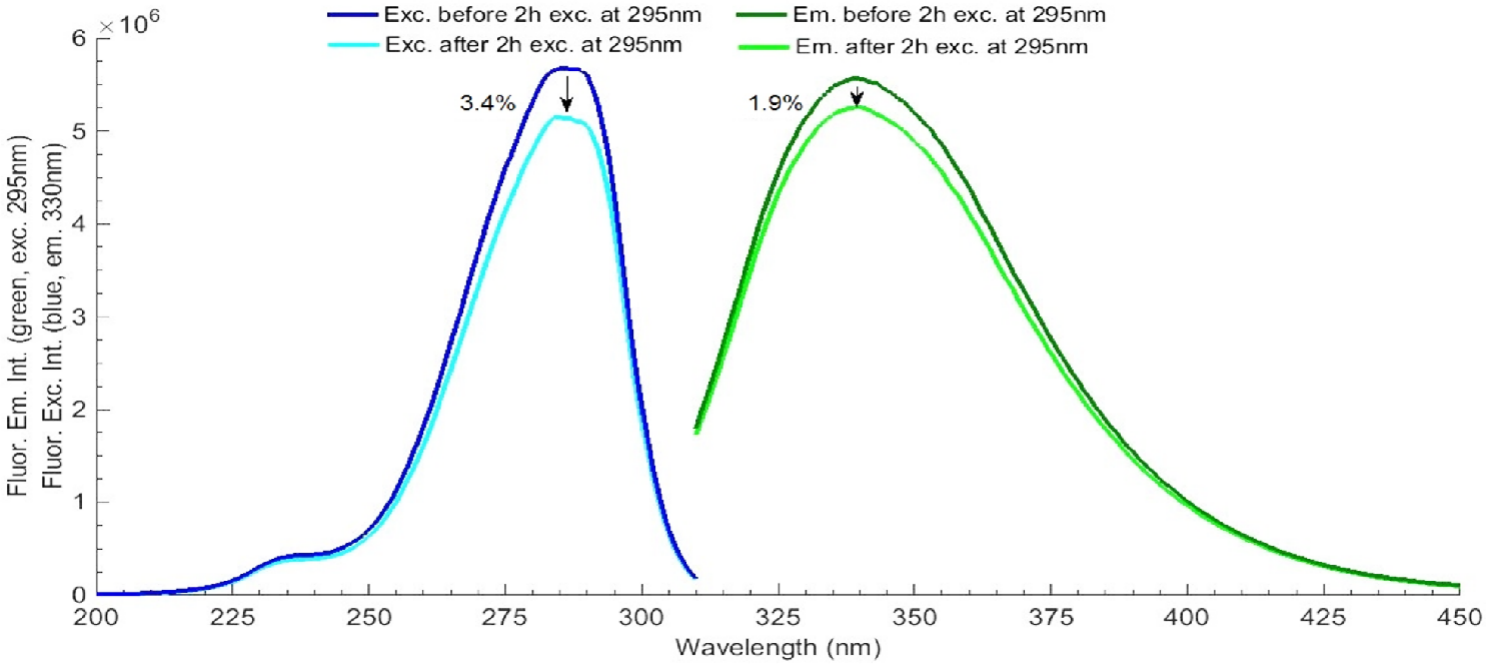
LYZ fluorescence excitation and emission spectra, before and after 295 nm continuous excitation for 2 hours. Fluorescence excitation was fixed at 295 nm and fluorescence emission at 330 nm and excitation slit was set at 0.1 mm (0.1 μW).

Continuous 295 nm illumination of LYZ (fresh sample, 10 μM) was carried out for 2 hours and the protein’s fluorescence emission intensity at 330 nm was monitored (see Fig 4). The excitation slit was set at 0.1 mm, with an equivalent lamp power of 0.1 μW, as in the thermal ramp experiment. The illumination spot was approximately 0.35 cm^2^. Irradiance was 0.343 W.cm^-2^. The excitation and emission spectra of LYZ prior and after the 2 hours continuous 295 nm excitation were analyzed (see Fig 5).

**Fig 6:**
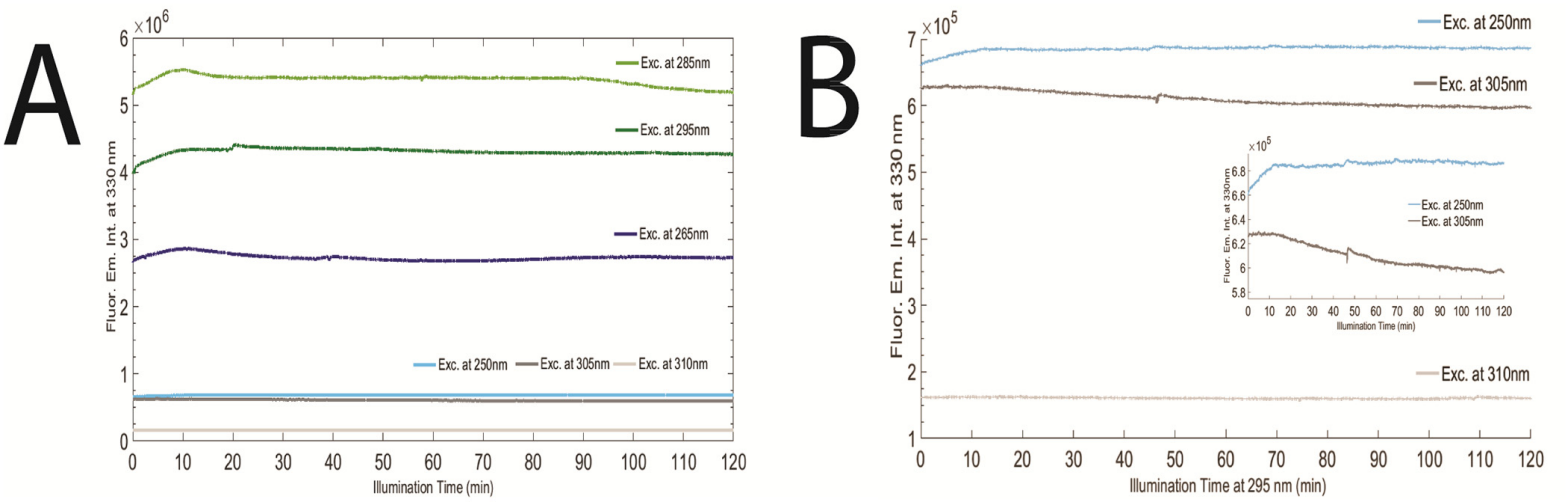
**A)** LYZ 2 hours excitation from 250 nm until 310 nm, at 20°C. Fluorescence emission wavelength was fixed at 330 nm, while excitation wavelengths were selected as following: at 250 nm (light blue), at 265 nm (dark blue), at 285 nm (light green), at 295 nm (dark green), at 305 nm (dark grey) and at 310 nm (light grey). **B)** Closer look at LYZ 2hours excitation at 250 nm, 305 nm and 310 nm, at 20°C. For all excitation wavelengths, fluorescence emission wavelength was fixed at 330 nm and slit fixed at 0.5 mm (1.0 μW).

The fluorescence emission intensity of LYZ at 330 nm was monitored upon continuous 2 hours excitation at six selected excitation wavelengths: 250 nm, 265 nm, 285 nm, 295 nm, 305 nm and 310 nm (see Fig 6). The excitation slit was fixed at 0.5 mm, corresponding to a 1.0 μW excitation power. The temperature of the solution was kept at 20°C using a Peltier element at the cuvette holder location. A fresh sample was used for each illumination run. The emission and excitation intensity values obtained were corrected in real-time for oscillations in the intensity of the excitation lamp.

#### SYPRO^®^ Orange for probing LYZ conformation changes

**Fig 7:**
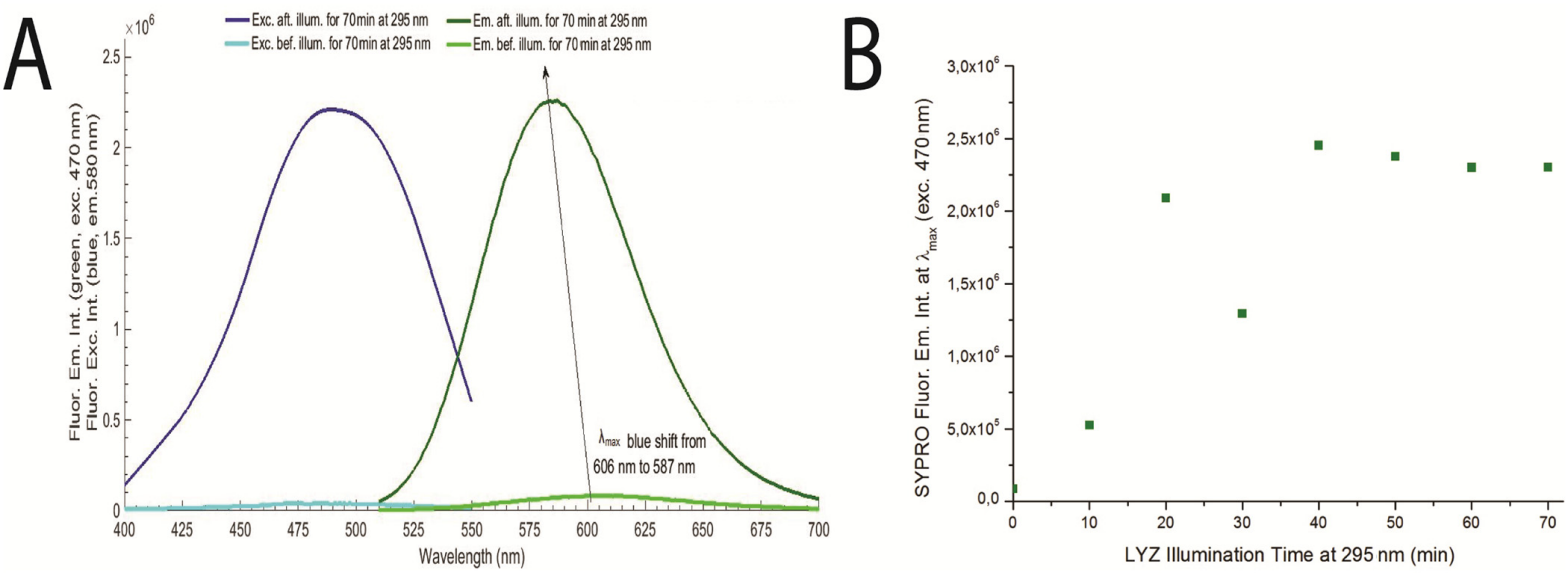
**A)** Monitoring LYZ with SYPRO^®^ Orange spectra before and after 70 min 295 nm excitation of LYZ at 20°C, with excitation slit fixed at 0.5 mm (1.0 μW). Fluorescence intensity of SYPRO^®^ Orange was fixed at 580 nm and excitation at 470 nm. **B)** SYPRO^®^ Orange emission intensity (excitation wavelength fixed at 470 nm) for every 10 min of LYZ 295 nm excitation for 70 min.

SYPRO^®^ Orange is used as a molecular probe in order to monitor protein conformational changes, since its fluorescence is greatly enhanced upon contact with hydrophobic environments [24]. A 3μL aliquot (dilution 1:1000) of SYPRO^®^ Orange stock solution (5,000X Concentrate in DMSO) was added to a cuvette containing a fresh sample of LYZ (10 μM, 3 mL) prior to the 295 nm continuous illumination experiment. The sample was gently shaken to mix both solutions. The fluorescence emission spectrum of SYPRO^®^ Orange, with excitation fixed at 470 nm and the fluorescence excitation spectrum of SYPRO^®^ Orange, with emission fixed at 580 nm, were acquired prior and after continuously illuminating LYZ at 295 nm for 70 min. Changes in LYZ fluorescence intensity and spectral shifts were quantified. The fluorescence emission intensity of SYPRO^®^ Orange at 580 nm (exc. at 470 nm) was monitored after every ten minutes of LYZ excitation at 295 nm for 70 min (see Fig 7). The excitation slit size was set at 0.5 mm (equivalent power lamp of 1.0 μW). The wavelength corresponding to the maximum fluorescence emission intensity of SYPRO^®^ Orange was monitored in order to detect the occurrence of possible spectral shifts during the illumination of LYZ at 295 nm (see Table 3).

**Table 3:** Wavelengths corresponding to maximum fluorescence emission intensity of SYPRO^®^ Orange after consecutive 10 min excitation cycles of LYZ at 295 nm.

Time (min)	λ_max_ (nm)	Δ(λ_t = 0_–λ_t = t_) (nm)
0	606	0
10	580	26
20	584	22
30	584	22
40	583	23
50	584	22
60	587	19
70	587	19

#### 295 nm excitation power effect on LYZ fluorescence emission

**Fig 8:**
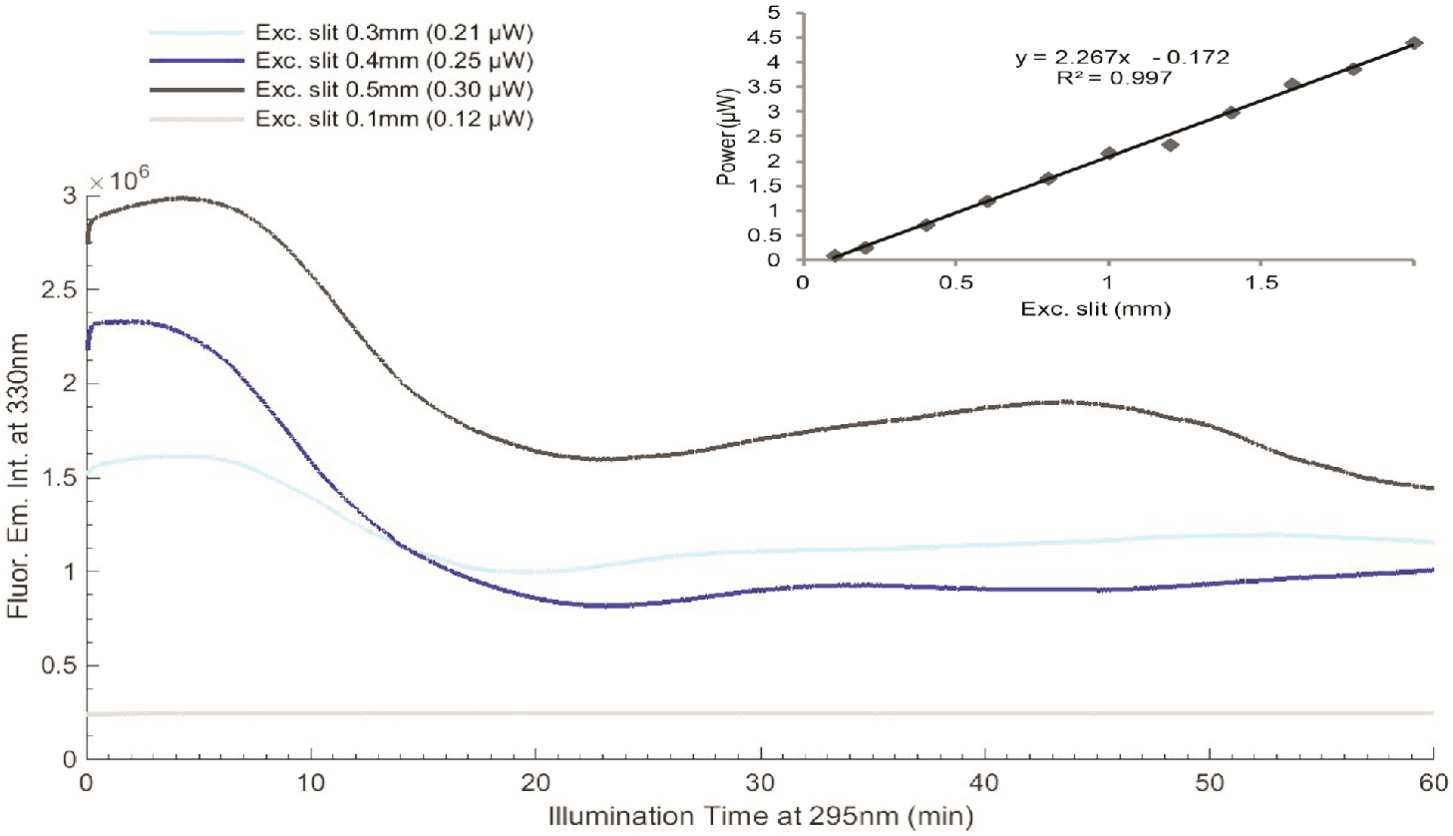
LYZ fluorescence 295 nm 2hours excitation at 20°C, using different excitation slit openings: 0.1 mm (0.1 μW), 0.3 mm (0.5 μW), 0.4 mm (0.7 μW) and 0.5 mm (1.0 μW). Fluorescence emission wavelength was fixed at 330 nm. At the upper corner is displayed the equation of the dependence of the excitation slit size (x) versus excitation power (y).

**Fig 9:**
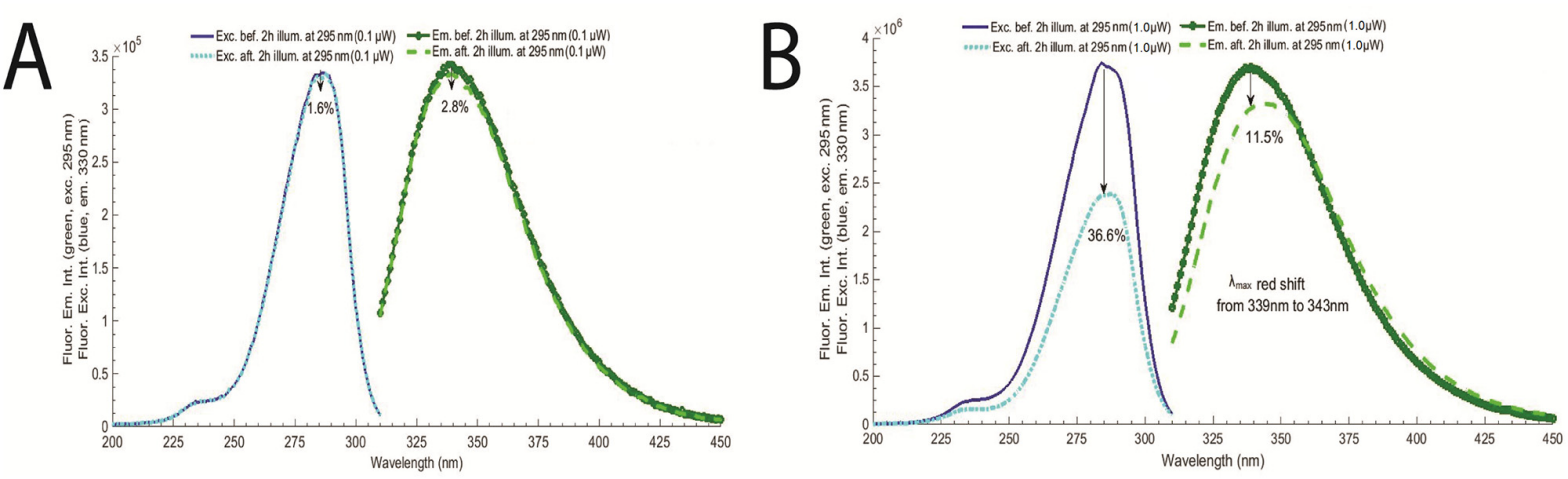
**A)** LYZ fluorescence excitation and emission spectra before and after LYZ 295 nm 2hours excitation, with slit opening fixed at 0.1 mm (0.1 μW). **B)** LYZ fluorescence excitation and emission spectra before and after LYZ 295 nm 2hours excitation, with slit opening fixed at 0.5 mm (power: 1.0 μW). Fluorescence excitation was fixed at 295 nm and fluorescence emission at 330 nm.

Firstly, the dependence of the excitation slit size versus excitation power was determined by measuring the power level at the cuvette location with a power meter (Ophir Photonics StarLite Meter ASSY ROHS, P/N7Z01565, Jerusalem, Israel), used with a power head (Ophir Photonics, 30A-BB-18 ROHS, P/N7Z02692, Jerusalem, Israel) upon varying the excitation slit size. The effect of 295 nm illumination power on the fluorescence emission intensity at 330 nm of LYZ was acquired using different excitation slit openings: 0.1 mm, 0.3 mm, 0.4 mm and 0.5 mm corresponding to 0.1 μW, 0.5 μW, 0.7 μW and 1.0 μW, respectively (see Fig 8). The temperature of the solution was kept at 20°C using a Peltier element at the cuvette holder location. A fresh sample was used for each illumination session. Fluorescence excitation (em. fixed at 330 nm) and emission (exc. fixed at 295 nm) spectra of LYZ were acquired before and after LYZ illumination with 0.1 mm and 0.5 mm excitation slit. The fluorescence intensity changes were quantified (see Fig 9).

#### Temperature effect on LYZ photochemistry

**Fig 10:**
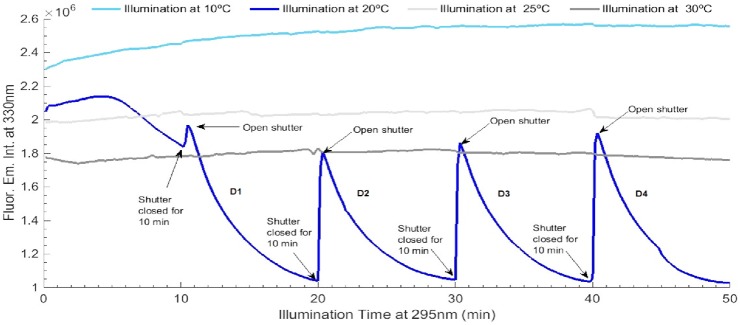
Temperature effect on LYZ photochemistry: at 10°C (light blue), 20°C (dark blue), 25°C (light grey) and 30°C (dark grey), for four open/close cycles of periods of 10 min of excitation followed by 10 min in the dark. Fluorescence excitation and emission wavelengths were fixed at 295 nm and 330 nm, respectively. Excitation slit size was set at 0.5 mm (1.0 μW) for all experiments.

The fluorescence emission intensity of LYZ at 330 nm was monitored upon 295 nm excitation. After 10 min of illumination the shutter was closed for another 10 min, remaining the protein in the dark. This excitation scheme was repeated four times. In total the protein was illuminated for 50 min and remained in the dark during 40 min. This experiment was repeated at four different temperatures: 10°C, 20°C, 25°C and 30°C. Four open/close cycles have been carried out. The excitation slit size was set at 0.5 mm (equivalent power lamp of 1.0 μW). A fresh sample was used for each experiment (see Fig 10).

**Fig 11:**
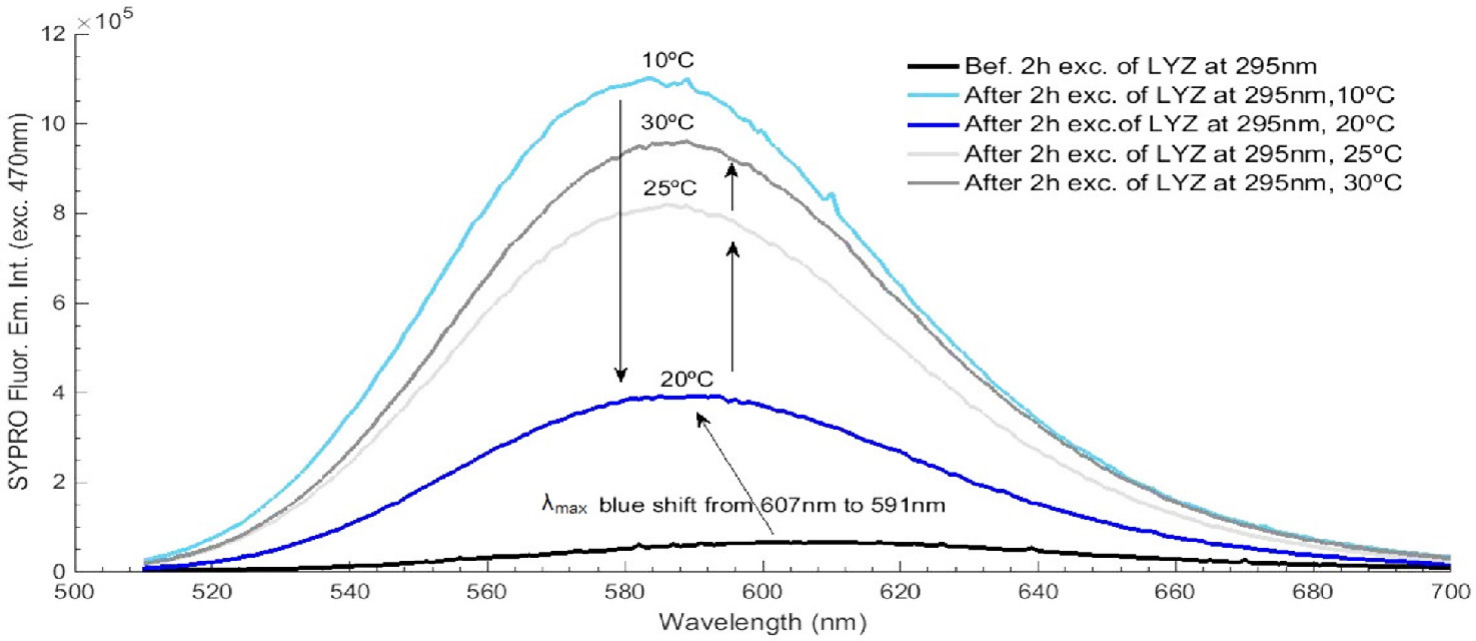
SYPRO^®^ Orange emission intensity spectra before and after LYZ 295 nm excitation at different temperatures (10°C, 20°C, 25°C and 30°C). SYPRO^®^ fluorescence excitation wavelength was fixed at 470 nm and acquired before and after the four open/close cycles. Excitation slit size was set at 0.5 mm (1.0 μW).

**Fig 12:**
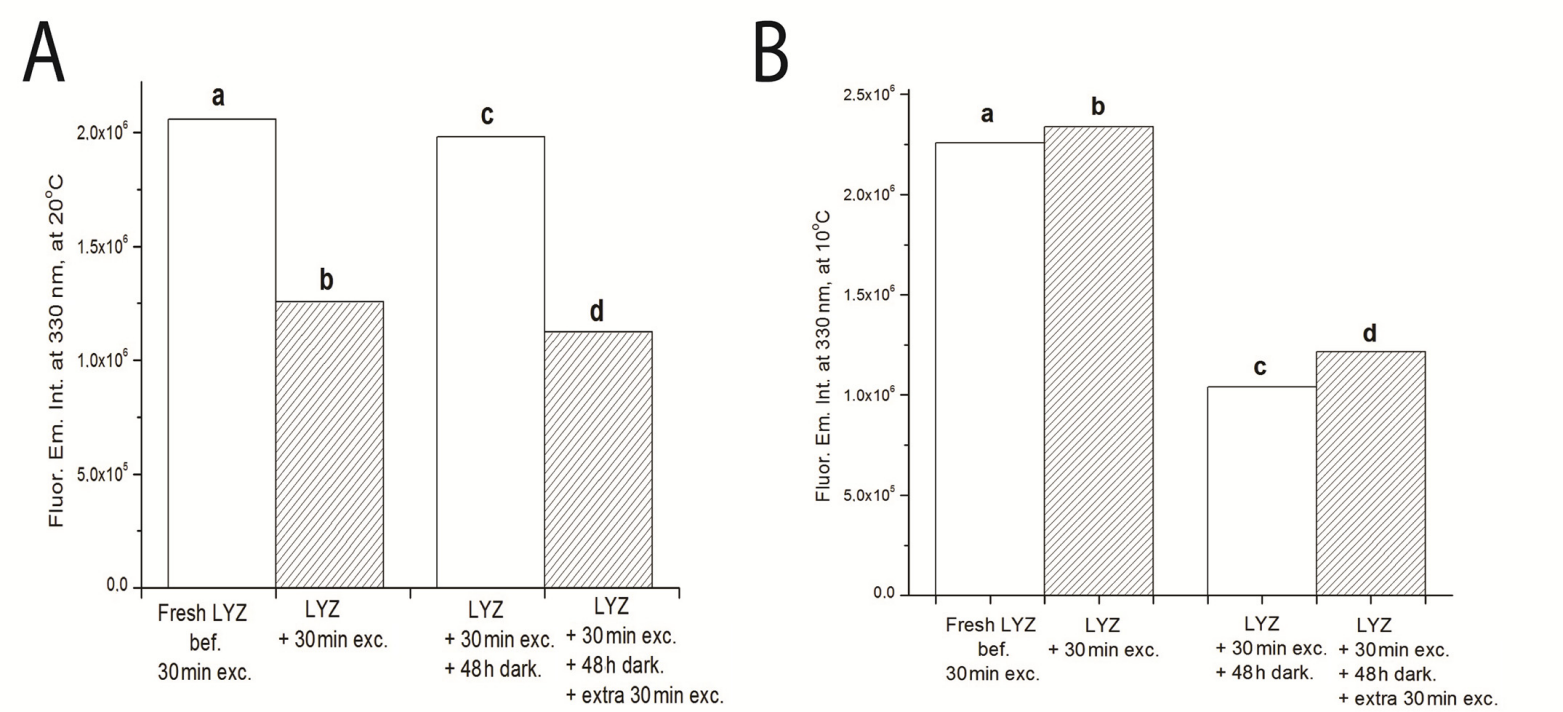
**A)** LYZ fluorescence emission intensity at 330nm monitored at 20°C; and **B)** at 10°C. Samples are described as: a) fresh LYZ never previously illuminated, b) LYZ after 30 min of continuous 295 nm illumination, c) LYZ after 30 min of continuous 295 nm illumination followed by 48 hours in the dark, d) LYZ after 30 min of continuous 295 nm illumination followed by 48 hours in the dark and subsequent further 30 min of continuous 295 nm. LYZ excitation was fixed at 295 nm with a slit size was set at 0.5 mm (1.0 μW).

SYPRO^®^ Orange was used in order to monitor putative LYZ conformational changes due to 295 nm illumination at different temperatures. Fluorescence emission spectra (exc. fixed at 470 nm) were acquired before and after the four open/close cycles (as described above) of 295 nm illumination of LYZ at each temperature (see Fig 11). Afterwards, the reversibility of the light induced processes at 10°C and 20°C were investigated. The fluorescence emission intensity at 330 nm of a four LYZ samples were monitored (exc. at 295 nm): a) fresh, non-illuminated sample, b) LYZ sample after 30 min of continuous 295 nm illumination (0.5mm slit size, with an equivalent power lamp of 0.30 μW), c) LYZ sample after 30 min of continuous 295 nm illumination followed by 48 hours in the dark and, d) LYZ sample after 30 min of continuous 295nm illumination followed by 48 hours in the dark and subsequent further 30 min of continuous 295 nm (see Fig 12).

#### Photochemistry of LYZ conjugated with HAOA gold nanoparticles

**Fig 13:**
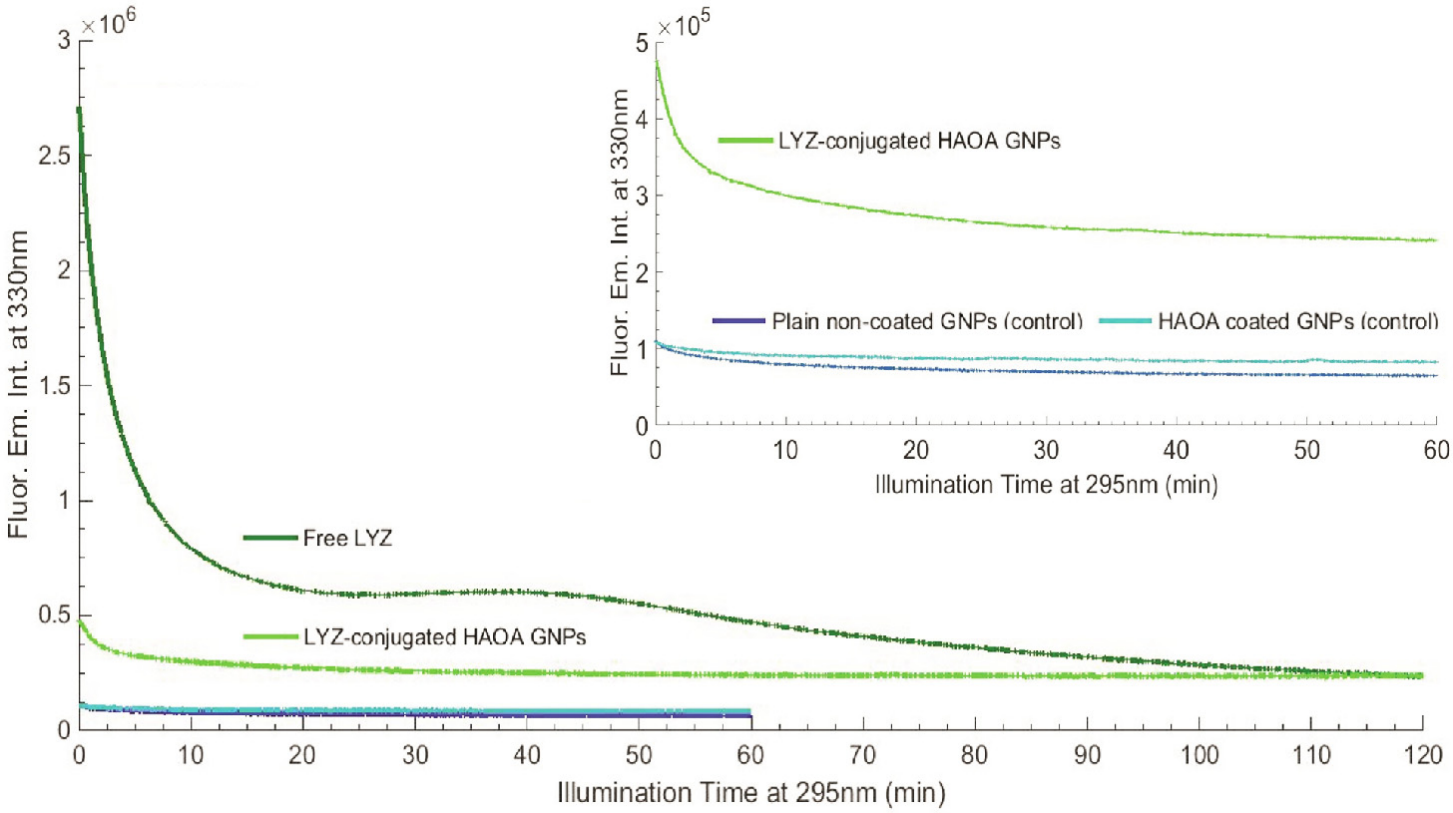
LYZ fluorescence emission intensity at 330 nm for free LYZ (2 h 295 nm excitation), LYZ-conjugated HAOA GNPs (2 h 295 nm excitation), and empty HAOA gold nanoparticles (GNPs) and non-coated plain GNPs (1h 295 nm excitation). All samples were analyzed at 20°C and excitation slit size fixed at 2.0 mm (4.4 μW). At the upper corner, LYZ-conjugated HAOA GNPs excited for 2 hours is compared to HAOA GNPs and plain GNPs.

**Fig 14:**
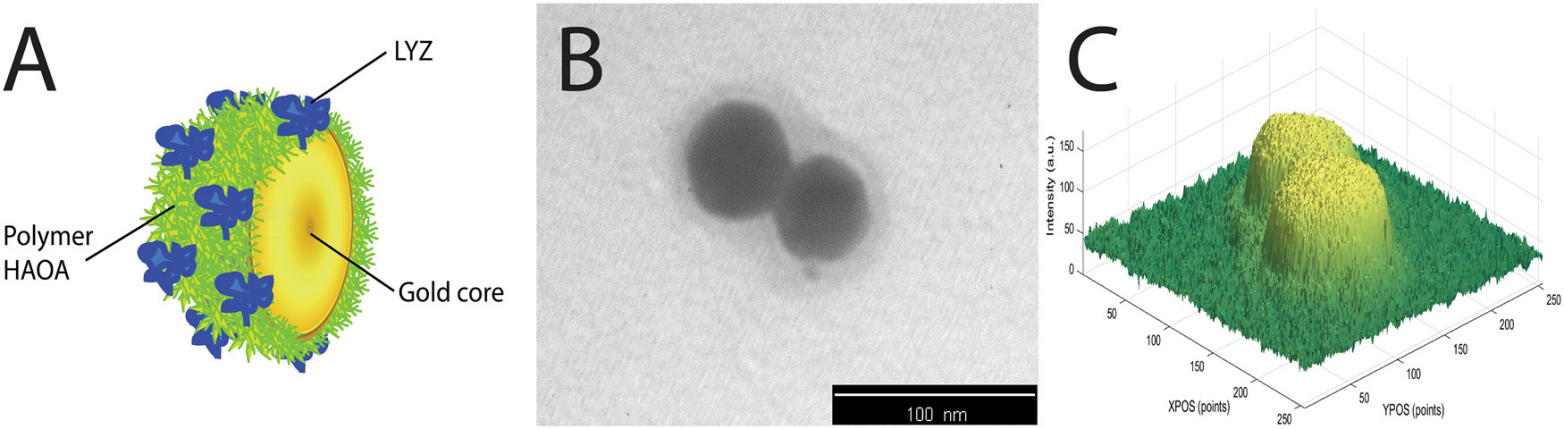
**A)** Representative illustration of LYZ-conjugated HAOA coated gold nanoparticles; **B)** TEM image of HAOA coated gold nanoparticles (non-conjugated) at scale bar: 100 nm; and **C)** Intensity analysis of the HAOA gold nanoparticles TEM image.

**Fig 15:**
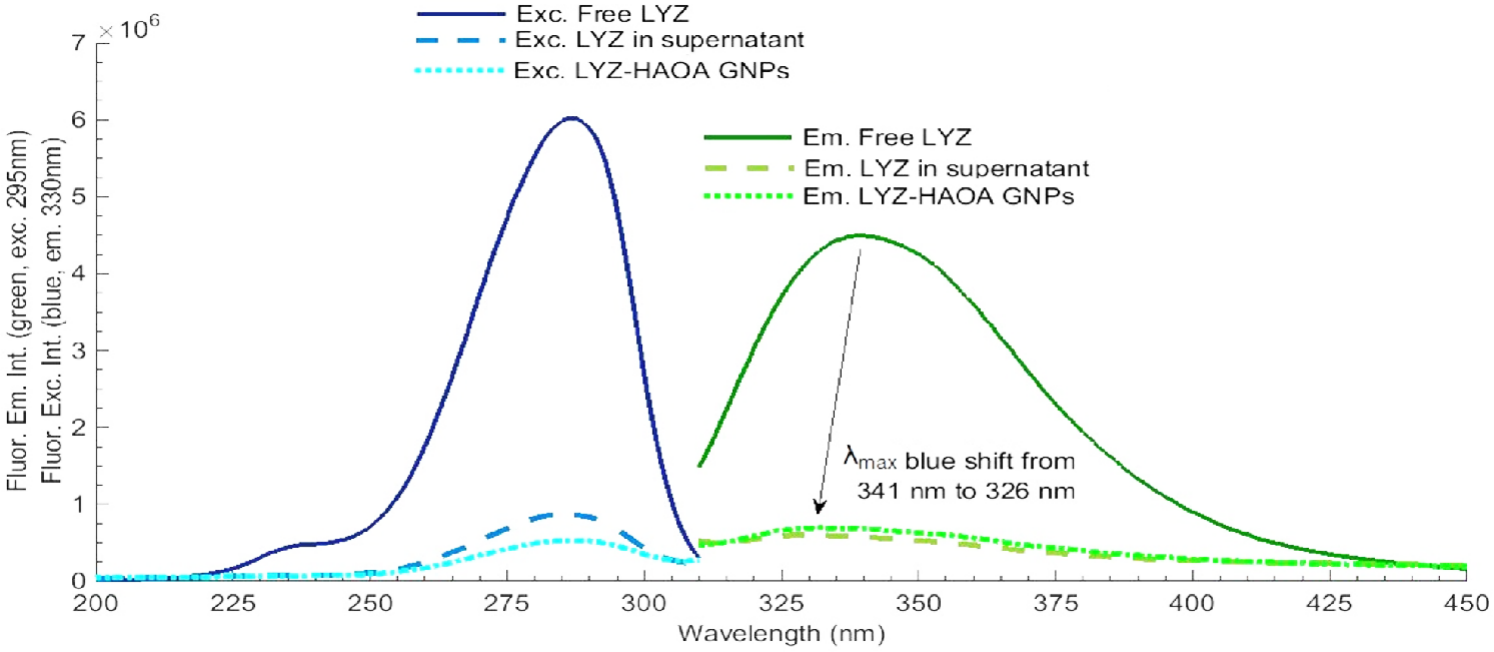
Conjugation effect: LYZ in supernatant (after conjugation) compared with free LYZ and LYZ-conjugated HAOA gold nanoparticles (GNPs). Fluorescence excitation spectra was fixed at 330 nm and fluorescence emission spectra was fixed at 295 nm. Experiments were conducted at 20°C and excitation slit size fixed at 2.0 mm (4.4 μW). LYZ was not continuously excited and only the necessary UV light was used for obtaining the displayed spectra.

**Fig 16:**
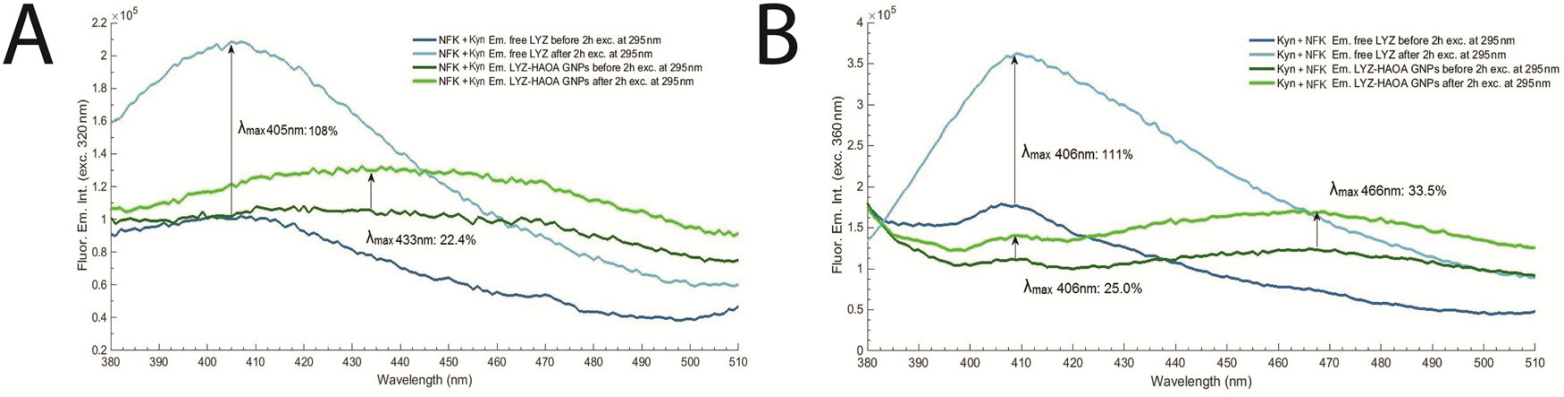
**A)** Fluorescence emission spectra for NFK + Kyn, before and after excitation of free LYZ and LYZ-conjugated HAOA gold nanoparticles (GNPs), at a fixed wavelength of 320 nm. Experiments were conducted at 20°C and excitation slit size fixed at 2.0 mm (4.4 μW); **B)** Fluorescence emission spectra for Kyn + NFK, before and after excitation of free LYZ and LYZ-conjugated HAOA gold nanoparticles (GNPs), at a fixed wavelength of 360 nm. Experiments were conducted at 20°C and excitation slit size fixed at 2.0 mm (4.4 μW).

The effect of continuous 295 nm excitation of LYZ has been investigated for LYZ conjugated to gold nanoparticles covered by natural polymers (hyaluronic acid, HA) and oleic acid (OA). Results have been compared with the data obtained with free LYZ (see Fig 13). Four samples have been continuously illuminated with 295 nm light for 2 hours at 20°C and their fluorescence emission intensity at 330 nm had been monitored: a) conjugated LYZ, b) free LYZ, c) plain non-coated gold nanoparticles and d) HAOA coated gold nanoparticles. The excitation slit was set at 2.0 mm (equivalent power of 4.4 μW at the sample location). The conjugation of LYZ onto the HAOA gold nanoparticles (see Fig 14) has been confirmed using steady state fluorescence spectroscopy. The fluorescence excitation (em. fixed at 330 nm) and emission (exc. fixed at 295 nm) spectra of non-conjugated LYZ, of the supernatant after centrifugation of the solution containing conjugated and non-conjugated LYZ, and of conjugated LYZ onto HAOA gold nanoparticles have been acquired in order to detect the presence of protein (see Fig 15). The formation of Trp photo degradation products upon 295 nm excitation of free LYZ and LYZ-conjugated HAOA gold nanoparticles has been confirmed using steady state fluorescence spectroscopy. In order to verify the formation of DT and NFK, fluorescence emission spectra were acquired upon 320 nm excitation of the solution before and after 2 hours of continuous excitation at 295 nm (see Fig 16A). In order to verify the presence of Kyn, emission spectra were obtained upon 360 nm excitation before and after 295 nm continuous excitation. Fluorescence changes have been quantified and compared for free and conjugated LYZ. A fresh sample was used for each illumination run (see Fig 16B).

### Time resolved fluorescence spectroscopy

The fluorescence lifetimes of free and conjugated LYZ have been acquired with fluorescence TCSPC lifetime spectrometer (DeltaPro, Horiba Scientific, Kyoto, Japan). A 280 nm and a 295 nm light emitting diode, <200 picoseconds FWHM with PPD and laser diode Horiba Scientific, Kyoto, Japan) was used to excite the samples. The fluorescence emission intensity at 330 nm was detected at the magic angle (54.7°) by a GaAs detector (Hamamatsu H7422P-40). The temperature of the solution was kept at 20°C using a Peltier element at the cuvette holder location. A fresh sample was used for each illumination run. A 300 nm long-pass filter (Semrock) was used in the emission channel. A solution of Ludox (colloidal silica) in Millipore water was used in order to acquire the instrument response function (IRF). Such response function has been used to deconvolve the protein decay. The decay times (τ) and pre-exponential factors (f_i_) recovered from the time resolved intensity decays for free LYZ and LYZ-conjugated HAOA gold nanoparticles at pH 7.4.

### HAOA gold nanoparticles physical characterization

The mean particle size, polydispersity index (PI) and zeta potential (ZP) for HAOA gold nanoparticles (non conjugated with LYZ) were also determined with a Coulter Nano-sizer Delsa Nano^™^C (Fullerton, CA). A low value of PI factor (< 0.25) indicates a more stable and less dispersed nanoparticles distribution in size. “D-value” was determined to describe the particle size distribution of 10%, 50% and 90% of the nanoparticles population.

### TEM analysis

HAOA gold nanoparticles structure and surface morphology were analyzed by Transmission Electron Microscopy (TEM, Zeiss M10, Germany). Samples were prepared through “sequential two-droplet” method by re-suspending the HAOA gold nanoparticles in distilled water and placing a drop (5–10 μL) of the suspension on to a formvar grid for 30–60 seconds. When the HAOA gold nanoparticles suspension had partly dried, the grid was washed three times with distilled water and the excess of water removed with a filter paper. Then, sodium phosphotungstate (PTA, 2%, w/v) was applied to the grid for 10 seconds, the excess of stain removed with a filter paper and the grid was left to dry at room temperature for 24 hours. Samples were analyzed at an accelerated voltage of 10–20 kV. Different fields of the images were recorded digitally, by using Matlab version R2014b (MathWorks, Massachusetts, USA), for determination of the intensity distribution of polymer HAOA and gold nanoparticles.

### Data Analysis

All data analysis, plotting and fitting procedures were done using Origin 8.1 (OriginLab Corporation, Northampton, MA, USA).

#### Emission Spectra and Excitation Spectra

Emission and excitation spectra were first smoothed using a 10 points adjacent averaging. All fluorescence spectra obtained were first Raman corrected by subtracting the spectra recorded for the buffer in solution. Normalized emission and excitation spectra were obtained by dividing each data point by the maximum intensity value in each spectrum.

### Fitting Procedures

#### LYZ fluorescence emission kinetic traces (em. at 330 nm) upon 295 nm continuous excitation

Each of the four decay curves observed upon 10 min of continuous 295 nm illumination after LYZ has been kept for 10 min in the dark at 20°C (see Fig 10), were fitted using a single exponential decay model given by the function *F(t) = C*_*1*_ *exp(-x/k_*1*_ *) + y*_*0*_. *F(t)* is the fluorescence emission intensity at 330 nm (a.u.) upon 295 nm at excitation time *t* (min), *y*_*0*_ and *C*_*1*_ are constants and *k*_*1*_ is the rate constant of fluorescence emission intensity decrease (min^-1^). *y*_*0*_ value was fixed to 0. The root mean square error R^2^ was > 0.99 for all traces. The fitted parameter values and corresponding errors, and root mean square error values obtained after fitting the 330 nm emission kinetic trace are displayed in Table 4.

**Table 4:** Single exponential fit using model *F(t) = C*_*1*_ *exp(-x/k_*1*_ *) + y*_*0*_ for each decay curve of LYZ at 20°C (D1, D2, D3 and D4; *D* stands for decay) (see Fig 10). Fit parameters are displayed in this table. Red. χ^2^ and Adj. R^2^ are reduced Qui-Square and Adjusted R- Square, respectively.

Decay curve	Parameters			Statistics	
	y_0_	C_1_	k_1_	Red. χ^2^	Adj. R^2^
D1	974297.8±3850.8	1.168E6±3954.1	21.1±0.2	4.61E7	0.999
D2	1.01E6±2172.9	887338.9±2627.9	18.9±0.2	2.22E7	0.999
D3	981472.1±2585.9	977520.3±2751.6	20.3±0.2	2.52E7	0.999
D4	949274.3±5964.5	1.09E6±6196.2	20.7±0.4	1.28E8	0.998

#### Free LYZ and LYZ conjugated HAOA gold nanoparticles fluorescence kinetics (em. at 330 nm) upon 295 nm excitation

The fluorescence emission intensity kinetic traces observed for free LYZ, plain non-coated gold nanoparticles, HAOA gold nanoparticles and LYZ-conjugated HAOA gold nanoparticles samples and displayed in Fig 13, after continuous 295 nm illumination for 2 hours, except for plain gold nanoparticles and HAOA gold nanoparticles without LYZ (60 min), at 20°C, were fitted using a double exponential decay model according to the formula *F(t) = y*_*0*_ *+C*_*1*_ *exp(-k_1_ *x)+C_*2*_ *exp(-k_2_ *x). *F(t)* is the fluorescence emission intensity at 330 nm (a.u.) upon 295 nm excitation at time *t* (min), *y*_*0*_, *C*_1_ and *C*_2_ are constants and *k*_1_ *and k*_2_ is the rate constant of fluorescence emission intensity decrease (min^-1^). *y*_*0*_ value was fixed to 0. The root mean square error R^2^ was > 0.99 for all kinetics. The fitted parameter values and corresponding errors, and root mean square error values obtained after fitting the 330nm emission kinetic trace are displayed in Table 5.

**Table 5:** Double exponential fit using model *F(t) = yo+C*_*1*_ *exp(-k_1_ *x)+C_*2*_ *exp(-k_2_ *x) for plain non-coated gold nanoparticles (GNPs), HAOA coated GNPs, free LYZ and LYZ-conjugated GNPs (see Fig 13).

Samples	Constants (y_0_, k_1_, k_2_)	R^2^	Pre-exponential factors (C_1_, C_2_)
**Free LYZ**	*For first 30min decay*:	0.999	C_1_ = 7.34E5 ± 7.10E3
	y_0_ = 5.84E5 ± 0.40E3		
	k_1_ = 1.42E-2 ± 0.20E-2		C_2_ = 1.42E6 ± 0.80E3
	k_2_ = 3.24E-3 ± 1.30E-5		
**LYZ—conjugated HAOA GNPs**	y_0_ = 9.83E4 ± 2.68E3	0.997	C_1_ = 1.44E4 ± 0.50E3
	k_1_ = 2.03E-3 ± 1.28E-4		
			C_2_ = 2.02E4 ± 2.14E3
	k_2_ = 1.22E-4 ± 2.78E-5		
**Plain non coated GNPs (control)**	y_0_ = 6.31E4 ± 9.00E1	0.998	C_1_ = 1.91E4 ± 1.70E2
	k_1_ = 6.20E-3 ± 1.09E-4		
			C_2_ = 2.46E4 ± 1.20E2
	k_2_ = 7.14E-4 ± 1.00E-5		
**HAOA coated GNPs (control)**	y_0_ = 7.93E4 ± 3.70E2	0.988	C_1_ = 1.40E4 ± 2.90E3
	k_1_ = 5.05E-3 ± 1.69E-4		
			C_2_ = 1.44E4 ± 2.6E3
	k_2_ = 4.23E-4 ± 2.83E-5		

#### Time resolved fluorescence

The fluorescence decay was analyzed by a routine based on the Marquardt least-squares minimization. The main equations for the time-resolved intensity decay data were assumed to be a sum of discrete exponentials as in:

$$F\left(t\right)=\sum_{i}\alpha_{i}.\text{exp}\left(-t/\tau_{i}\right)$$

where *F*(*t*) is the intensity decay, α_*i*_ is the amplitude (pre-exponential factor), τ_*i*_ the fluorescence lifetime of the *i*-th discrete component, and ∑ατ_*i*_ = 1.0.

The fractional intensity *fi* of each decay time is given by:

$$f_{i}=\frac{\alpha_{i}\tau_{i}}{\sum_{i}\alpha_{i}\tau_{i}}$$

and the mean lifetime is:

$$\langle \tau \rangle =\sum_{i}f_{i}\tau_{i}$$

The fluorescence lifetimes of free LYZ and LYZ-conjugated HAOA gold nanoparticles acquired upon 280 nm and 295 nm excitation are summarized in Table 6.

**Table 6:** Recovered fluorescence lifetimes (τ_i_), pre-exponential factors (α_i_), intensity fraction (f_i_) and average lifetime (<τ>) for free LYZ and LYZ-conjugated HAOA gold nanoparticles (GNPs), at pH 7.4 obtained by a nonlinear fit using the PTI software. Excitation has been carried out using a 280 nm and 295 nm diodes.

Lifetime (ns)		Intensity fraction		Pre-exponential factor	
**Free LYZ (280 nm diode)**					
τ_1_	0.41±0.03	f_1_	0.21	α_1_	1.02±0.04
τ_2_	1.53±0.08	f_2_	0.48	α_2_	0.63±0.02
τ_3_	3.33±0.13	f_3_	0.31	α_3_	0.19±0.03
<τ>	1.86				
**LYZ-conjugated HAOA GNPs (280 nm diode)**					
τ_1_	0.17±0.008	f_1_	0.50	α_1_	3.97±0.16
τ_2_	1.58±0.04	f_2_	0.36	α_2_	0.31±0.01
τ_3_	9.20±0.76	f_3_	0.14	α_3_	0.02±0.002
<τ>	1.91				
**Free LYZ (295 nm diode)**					
τ_1_	0.27±0.02	f_1_	0.26	α_1_	1.85±0.06
τ_2_	1.05±0.05	f_2_	0.41	α_2_	0.74±0.04
τ_3_	3.02±0.06	f_3_	0.33	α_3_	0.21±0.01
<τ>	1.50				
**LYZ- conjugated HAOA GNPs (295 nm diode)**					
τ_1_	0.046±0.009	f_1_	0.41	α_1_	9.62±1.21
τ_2_	0.38±0.01	f_2_	0.50	α_2_	1.44±0.08
τ_3_	2.77±0.38	f_3_	0.03	α_3_	0.01±0.002
τ_4_	36.0±6.30	f_4_	0.06	α_4_	0.002±0.0002
<τ>	1.43				

## Results

In Table 1 are listed the absorption and fluorescence spectral characteristics of N-formylkynurenine (NFK), dityrosine (DT) and kynurenine (Kyn) [25–27].

In Fig 1 is displayed the 3D molecular structure of hen egg white LYZ (2LYZ.pdb), highlighting Trp and Tyr residues and disulphide bridges. Trp residues are displayed in red, Tyr residues in blue and Cystines in yellow. LYZ has in total 6 Trp residues, 3 Tyr residues and 4 disulphide bridges. In Table 2 are listed the shortest distances between each Trp and Tyr residues and the nearby disulphide (SS) bonds. The shortest distance between Trp123 and SS bridge C30-C115 is 3.326 Å. All considered distances were < 12 Å.

In Fig 2 is displayed the fluorescence emission intensity at 350 nm, upon continuous excitation at 295 nm (0.1 μW) of a 10 μM LYZ solution as a function of temperature, from 45°C and 90°C. The heating rate was fixed at 1°C/min. The first derivative is represented in the upper right corner. The melting temperature (T_m_) of LYZ was determined to be 74°C, consistent with literature values obtained under similar physicochemical conditions [28, 29]. The fluorescence emission intensity at 350 nm decreased from 2.4E6 to 1.1E6 counts (56%) during the thermal scan from 45–90°C.

The fluorescence excitation and emission spectra of LYZ, before and after thermal unfolding, are displayed in Fig 3. While acquiring the fluorescence excitation spectra the emission was fixed at 350 nm. The fluorescence emission spectra were acquired upon 295 nm excitation. After thermal unfolding, the fluorescence emission intensity of LYZ at the wavelength where maximum emission is observed (340 nm) has decreased by 73% and the fluorescence excitation intensity at the wavelength where maximum excitation is observed (285 nm) has decreased by 64%. Furthermore, the fluorescence emission spectrum of LYZ has been red shifted from 340 nm to 349 nm, after protein unfolding.

In Fig 4 is displayed the fluorescence emission intensity kinetic trace at 330 nm upon continuous illumination of a fresh LYZ sample (10 μM) at 295 nm, for 2 hours with a excitation slit of 0.1 mm (equivalent power: 0.1 μW). Two transitions are visible: the first one at 10 min and the second one, smaller, at 90 min. The fluorescence emission intensity increases 1.0% in the initial 10 min of illumination, followed by a decay in the fluorescence emission intensity until 70 min, reaching an intensity level lower than the initial one. Afterwards, the fluorescence emission intensity increases peaking around 90–95 min of illumination and stabilizes thereafter.

The fluorescence excitation and emission spectra of LYZ, before and after 120 min of continuous 295 nm illumination (excitation slit at 0.1 mm, 0.1 μW) are displayed in Fig 5. While acquiring the fluorescence excitation spectra the emission was fixed at 330 nm. The fluorescence emission spectra were acquired upon 295 nm excitation. After 2 hours of continuous 295 nm excitation the fluorescence emission intensity of LYZ at 330 nm decreased 3.4% and the fluorescence excitation intensity decreased 1.9%. No shift has been observed in the fluorescence emission spectrum. The same spectra were acquired after 2 hours of excitation at 295 nm using a larger excitation power of 1.0 μW (exc. slit 0.5 mm) (data not shown). Both spectra show that the effect of 295 nm continuous excitation on the fluorescence emission and excitation spectra of LYZ is smaller than the effect of thermal unfolding.

In Fig 6 (panel A and B) is displayed the fluorescence emission of LYZ at 330 nm upon continuous 2 hours illumination, at 20°C, at six selected wavelengths: 250 nm, 265 nm, 285 nm, 295 nm, 305 nm and 310 nm. At 250 nm, 265 nm, 285 nm and 295 nm the kinetic traces are similar. An initial increase in the fluorescence emission intensity is followed by a plateau like region with smaller oscillations. The initial fluorescence emission intensity upon 250 nm, 265 nm, 285 nm and 295 nm illumination increases by 3.7%, 6.9%, 6.7%, 8.6%, respectively. Continuous illumination with 305 nm light leads to a 4.7% decrease in the fluorescence emission intensity after 2 hours of continuous illumination (Fig 6, panel B). Continuous illumination with 310 nm light does not lead to fluorescence emission intensity changes.

In Fig 7A is displayed the fluorescence emission spectrum of SYPRO^®^ Orange, upon 470 nm excitation and the fluorescence excitation spectrum of SYPRO^®^ Orange, with emission fixed at 580 nm, prior and after continuously illuminating LYZ at 295 nm for 70 min (1.0 μW). SYPRO^®^ Orange fluorescence emission intensity at the wavelength where maximum emission is observed (580 nm) has increased by 25.5-fold and the fluorescence excitation intensity at the wavelength where maximum excitation is observed (470 nm) has increased by 52.3-fold. Furthermore, the fluorescence emission spectrum of SYPRO^®^ Orange exhibits a 19 nm blue shift after 295 nm continuous excitation of LYZ. The wavelength at maximum SYPRO^®^ Orange fluorescence emission intensity has shifted from 606 nm to 587 nm after 70 min of illumination. As a control, the fluorescence emission of SYPRO^®^ Orange at 580 nm, upon excitation at 470 nm, was monitored after SYPRO^®^ Orange has been continuously illuminated at 295 nm for 70 min. A bleaching effect of 18% was observed (data not shown), and no blue shift was observed.

LYZ was exposed to 10min continuous 295 nm excitation cycles (0.5 mm; 1.0 μW). After each excitation period, the fluorescence excitation and emission spectra of SYPRO^®^ Orange were acquired. In total, LYZ was excited with 295 nm light for 70 min. In Fig 7B is displayed the fluorescence emission intensity of SYPRO^®^ Orange (upon exc. at 470 nm) after each 10 min excitation period of LYZ at 295 nm. The SYPRO^®^ Orange emission intensity is observed to increase 23.6-fold after the initial 20 min of LYZ excitation, followed by a decrease in the third round of LYZ excitation. Afterwards, SYPRO^®^ Orange fluorescence emission intensity increases once again and stabilizes after 40 min of 295 nm excitation. Wavelengths corresponding to the maximum fluorescence emission intensity of SYPRO^®^ as a function of LYZ illumination time are displayed in Table 3.

The effect of 295 nm excitation power on the fluorescence emission profile of LYZ is displayed in Fig 8. The power has been controlled using different excitation slit openings: 0.1 mm, 0.3 mm, 0.4 mm and 0.5 mm corresponding to 0.1 μW, 0.5 μW, 0.7 μW and 1.0 μW, respectively. The equation displaying the dependence of the excitation slit size (x) versus excitation power (y) was found to be y = 2.267x − 0.172, with R^2^ = 0.997 (see insert in Fig 8). At the lower power level (0.1 μW) almost no changes are observed in the fluorescence emission intensity of LYZ upon continuous excitation at 295 nm. For all other power levels, an initial increase in the fluorescence emission intensity is observed, followed by a decrease in intensity and subsequent weak increase.

The effect of increasing the power from 0.1 μW to 1.0 μW (exc. slit increase from 0.1 mm to 0.5 mm) in the fluorescence emission and excitation spectra of LYZ after 2 hours of continuous 295 nm excitation is displayed in Fig 9 (panels A and B, respectively). When carrying out the illumination with 0.1 μW, a slight decrease in the excitation and emission intensity spectra is observed. At the wavelength of maximum fluorescence intensity, the fluorescence excitation and emission intensities dropped 1.6% and 2.8%, respectively (Fig 9A). When carrying out the illumination with 1.0 μW, the fluorescence excitation and emission intensities dropped 36.6% and 11.5%, respectively, at the wavelength of maximum fluorescence intensity (Fig 9B).

In Fig 10 is displayed the fluorescence emission intensity of LYZ at 330 nm upon non continuous 295 nm illumination for 90 min at four different temperatures: 10°C, 20°C, 25°C and 30°C. After each 10 min excitation period, the shutter has been closed for 10 min, followed by another 10 min excitation at 295 nm. Four open/close cycles have been carried out. The observed kinetic traces are very dependent on temperature. At 10°C, LYZ fluorescence emission intensity increases by 11% after 90 min (a total of 50 min of non continuous excitation and a total of 40 min in the dark). The periods of darkness do not lead to changes in the fluorescence emission intensity of LYZ when the new 10 min excitation cycle starts. At 20°C, LYZ shows a very different response: the protein’s fluorescence emission intensity decreases exponentially upon continuous excitation at 295 nm, except during the first 10 min where the fluorescence emission intensity increases during the first 5 min prior to decreasing. A 49.7% drop in fluorescence emission intensity is observed after the four last 10 min excitation cycles. After being kept in the dark for 10 min, LYZ recovers the lost fluorescence emission intensity. This experiment was repeated three times in order to confirm its reproducibility and the reversibility of the process (data not shown). Every time fluorescence recovery was observed at 20°C. Each of the four decay curves observed after the initial 10 min of excitation have been fitted by a single exponential decay model given by y = C_1_*exp(-x/k_1_) + y_0_. The fitted parameter values (C_1_, k_1_, y_0_) and corresponding errors, as well as root mean square error values, were obtained after fitting each kinetic trace. The results are displayed in Table 4. At last, continuous 295 nm excitation of LYZ at 25°C and 30°C does not significantly change the fluorescence emission intensity of the protein.

SYPRO^®^ Orange was used to monitor possible conformational changes of LYZ at the end of the excitation/darkness cycles described above. In Fig 11 is displayed the SYPRO^®^ Orange fluorescence emission intensity spectra, upon 470 nm excitation, acquired before and after the full 295 nm illumination of LYZ at each temperature as shown in Fig 10. SYPRO^®^ Orange fluorescence emission intensity increased after each experiment. An intensity increase of 15.1-fold, 4.6-fold, 11.1-fold and 14.6-fold is observed at 10°C, 20°C, 25°C, and 30°C, respectively. The larger fluorescence emission intensity increase is observed at 10°C and the smallest fluorescence increase is observed at 20°C. Furthermore, the wavelength at maximum fluorescence emission intensity of SYPRO^®^ Orange is observed to be 16 nm blue shifted from 607 nm to 591 nm after each experiment.

In order to confirm the observed reversibility of the process at 20°C and the lack of reversibility at 10°C the fluorescence emission intensity at 330 nm of the following samples has been monitored at 20°C and 10°C (Fig 12, panels A and B, respectively): a) fresh LYZ never previously illuminated, b) LYZ after 30 min of continuous 295 nm illumination, c) LYZ after 30 min of continuous 295 nm illumination followed by 48 hours in the dark, d) LYZ after 30 min of continuous 295 nm illumination followed by 48 hours in the dark and subsequent further 30 min of continuous 295 nm. The results are presented in Fig 12 (panels A and B display the data acquired at 20°C and at 10°C, respectively). It can be seen that LYZ has lost fluorescence emission intensity when illuminated for 30 min at 295 nm. However, it has recovered its original fluorescence emission intensity after left in the dark for 48 hours, confirming the results at 20°C displayed in Fig 10. On the other hand, at 10°C it can be observed that continuous 295 nm excitation leads to a slight increase of protein fluorescence emission, as displayed in Fig 10. However, 48 hours in the dark did not lead to the recovery of the initial fluorescence emission intensity values.

The response of conjugated LYZ and non-conjugated LYZ to 295 nm continuous excitation has been investigated in order to infer the possible protective effect of gold nanoparticles covered by natural polymers (hyaluronic acid, HA) and oleic acid (OA) towards photochemistry. LYZ has been conjugated to HAOA coated gold nanoparticles. The fluorescence emission intensity at 330 nm as a function of continuous 295 nm excitation of conjugated LYZ, free LYZ, plain non-coated gold nanoparticles and HAOA coated gold nanoparticles is displayed in Fig 13. The initial fluorescence emission of conjugated LYZ is quenched by one order of magnitude when compared to the initial fluorescence emission intensity of free LYZ. After 2 hours of continuous 295 nm excitation the fluorescence emission intensity of free LYZ has dropped by 91.3% while the fluorescence emission intensity of conjugated LYZ has only dropped by 50.0%. Interestingly, both samples end up with the same fluorescence intensity level after 2 hours excitation. Both kinetic traces have been fitted with a double exponential decay model according to the formula *F(t) = y*_*0*_ *+C*_*1*_ *exp(-k_1_ *x)+C_*2*_ *exp(-k_2_ *x). In Table 5 are displayed the fitting parameters and corresponding errors as well as the root mean square error values for double exponential decay curves. For free LYZ, k_1_ and k_2_ were, respectively, 1.42E-2 ± 0.20E-2 and 3.24E-3 ± 1.30E-5 (for the first 30 min of the decay), while for LYZ-conjugated HAOA coated gold nanoparticles, k_1_ and k_2_ were, respectively, k_1_ = 2.03E-3 ± 1.28E-4 and k_2_ = 1.22E-4 ± 2.78E-5. We could confirm the protective effect of HAOA coated gold nanoparticles towards LYZ since the decay constants for conjugated LYZ were lower compared to the ones for free LYZ. The fluorescence emission intensities at 330 nm displayed by the plain or HAOA coated gold nanoparticles are only residual and do not change significantly upon continuous 295 nm illumination.

In Fig 14A is displayed a graphical representation of the HAOA coated gold nanoparticles. We have confirmed the HAOA gold nanoparticles structure and coating formation by TEM, as represented in Fig 14B. The mean particle size and polydispersity index (PI) for HAOA gold nanoparticles (non-conjugated with LYZ) was also determined to be around 300 nm (PI: 0.2) and a zeta potential (ZP) of -19 mV. However, the volume distribution for 90% of HAOA gold nanoparticles (D90%) was higher for smaller sized particles (D90% = 149 nm), as confirmed in TEM analysis. Here we observe that HAOA gold nanoparticles show a size around 50–100 nm. This variation could be explained by the presence of some aggregated in suspension. Finally, we have analyzed the image intensity by using Matlab version R2014b (MathWorks), as plotted in Fig 14C. We can observe that there is a core substrate, which is probably the gold core of the nanoparticles, while on top of this substrate there is a matrix, which we assign to the polymer HAOA distributed around the gold nanoparticles.

In Fig 15 is displayed the fluorescence excitation (emission at 330 nm) and emission (excitation at 295 nm) spectra of free LYZ, of the supernatant after centrifugation of the solution containing conjugated and free LYZ, and of conjugated LYZ HAOA gold nanoparticles. Data confirm the conjugation of LYZ onto the HAOA gold nanoparticles. Free LYZ shows the highest fluorescence excitation intensity, followed by LYZ present in the supernatant (that didn’t conjugate with HAOA gold nanoparticles) and, finally, LYZ conjugated HAOA gold nanoparticles. In the case of the fluorescence emission spectra, the emission intensity of LYZ conjugated HAOA gold nanoparticles was slightly higher than free LYZ in the supernatant, after nanoparticles isolation.

In Fig 16A is displayed the fluorescence emission spectra of four samples upon excitation at 320 nm: a) non-illuminated free LYZ, b) free LYZ excited with 295nm for 2 hours, c) non-illuminated LYZ-HAOA gold nanoparticles, d) LYZ-HAOA gold nanoparticles excited with 295 nm for 2 hours. The 320 nm excitation is intended to detect the presence of NFK and DT, a Trp and Tyr photoproduct, respectively. However, 320 nm light can still excite Kyn, despite its maximal excitation at 360 nm. For the free enzyme, a peak centred at 405 nm is observed upon 320 nm excitation. The fluorescence emission intensity of the peak increases 108% after continuous excitation with 295 nm for 2 hours. For the conjugated enzyme, a broad peak centred at 433 nm is observed. After continuous excitation with 295 nm for 2 hours, the fluorescence emission intensity is observed to increase by 22.4% for the peak centred at 433 nm.

In Fig 16B is displayed the fluorescence emission intensity spectra of four samples upon excitation at 360 nm: a) non-illuminated free LYZ, b) free LYZ excited with 295 nm for 2 hours, c) non-illuminated LYZ-HAOA gold nanoparticles, d) LYZ-HAOA gold nanoparticles excited with 295 nm for 2 hours. The 360 nm excitation is intended to excite Kyn, a Trp photoproduct. However, this wavelength will also excite NFK but not excite DT. For the free enzyme, a peak centred at 406 nm is observed upon 360 nm excitation. The fluorescence emission intensity of the peak increases 111% after continuous excitation with 295 nm for 2 hours. For the conjugated enzyme, two main peaks are observed: one centred at 406 nm and another centred at 466 nm. After 295 nm excitation for 2 hours, the fluorescence emission intensity is observed to increase by 25.0% and by 33.5% for the peaks centred at 406 nm and 466 nm, respectively.

In Table 6 are displayed the fluorescence lifetimes (τ_*i*_) distribution and the associated pre-exponential factors (f_*i*_) recovered from the time resolved intensity decays for non-illuminated LYZ and LYZ-conjugated HAOA gold nanoparticles (diode excitation wavelengths: 280 nm and 295 nm; emission wavelength: 330 nm) at pH 7.4. The structural changes induced in free LYZ and conjugated LYZ are visible in fluorescence lifetime distribution, as the lifetime for the free LYZ is shorter than for LYZ-conjugated HAOA gold nanoparticles, for both diodes studies. For free LYZ, at 280 nm and 295 nm, 3 lifetimes are present, as well as for the LYZ-conjugated HAOA gold nanoparticles, except for LYZ-conjugated HAOA gold nanoparticles at 295 nm with 4 lifetimes.

## Discussion

The structure of lysozyme (LYZ) has been well characterized in literature [13, 23, 30, 31]. Its 3D structure displayed in Fig 1 shows the presence of four disulphide bonds (SS) in close spatial proximity to six Trp residues (Table 2). As previously published by Neves-Petersen et al. [8, 14, 15], excitation of the side chains of aromatic residues located in close spatial proximity to SS bridges may lead to the disruption of the SS bonds. SS bridges are known to be excellent quenchers of aromatic residues. Therefore, when the bridges are broken, Trp fluorescence intensity might increase [8]. The observed result will depend on the nature of the photochemical pathways that the protein enters after excitation (*vide infra*).

In this paper, we have investigated the effect of temperature and of 295 nm continuous excitation on LYZ. LYZ melting temperature has been determined with temperature dependent steady-state fluorescence measurements and was found to be ~74°C, at pH 6.0 (Fig 2), which is close to the value reported by other research groups when carrying out thermal unfolding of LYZ at similar conditions [28, 29]. After thermal unfolding, the fluorescence emission of LYZ is observed to be 9 nm red-shifted, indicating that the Trp moiety in LYZ became more solvent accessible (Fig 3). Trp fluorescence emission is known to be very sensitive to the dielectric constant and therefore to the polarity of the medium surrounding it. In polar environments the fluorescence emission is red shifted compared to Trp emission in apolar environments. Solvent relaxation prior to fluorescence emission is responsible for this observation. The drop in excitation and emission intensity of LYZ observed after thermal unfolding (64% and 73%, respectively) reveals that the Trp moiety is now more quenched due to solvent exposure and/or that upon 295 nm excitation some of the Trp residues have been converted into photoproducts, such as NFK and Kyn (see Table 1), which have their excitation and emission spectra shifted with respect to Trp. The latter is confirmed by the data displayed in Fig 16, which shows that NFK and Kyn are formed upon 295 nm excitation of LYZ.

During the thermal unfolding experiment (Fig 2) the protein has also been exposed to 295 nm light. The results of such experiment (Figs 2 and 3) are a combined effect of temperature and UV exposure. To minimize the effect of UV light on the observed results a small excitation slit opening of 0.1 mm has been selected. In order to investigate the sole effect of 295 nm light on the observed fluorescence changes, LYZ has been continuously excited by 295 nm light for 2 hours, at 20°C, using an excitation slit opening of 0.1 mm (0.1 μW) (Fig 4). After 2 hours illumination, the fluorescence excitation and emission intensities at the respective wavelength corresponding to maximum intensity have decreased by only 3.4% and 1.9%, respectively and no spectral shift was observed (Fig 5). It is likely that these changes would be even smaller upon 45 min continuous excitation at 295 nm (the illumination time used during the thermal unfolding assay). This shows that the effect of temperature during the thermal unfolding experiment exceeds the effect of 295 nm illumination when a 0.1 mm slit is used. This also confirms that thermal unfolding studies should always be carried out using low excitation power in order to minimize photochemistry, which will influence the recovered T_m_ value.

The time dependent fluorescence emission kinetic traces of LYZ acquired upon continuous illumination with different UV wavelengths (250 nm, 265 nm, 285 nm, 295 nm, 305 nm and 310 nm) at 20°C are displayed in Fig 6. Excitation at 250 nm is known to cause direct photolysis of SS bonds [12]. 250 nm also excites Trp residues but marginally excites Tyr and Phe residues (see Table 7). This means that excitation of LYZ with 250 nm will lead to the disruption of SS bridges, according to literature [32–35]. In addition, 265 nm excites Tyr and Trp and marginally excites Phe, 285 nm excites Trp and Tyr, 295 nm excites Trp residues and very marginally excites Tyr residues, and finally, 305 nm and 310 nm marginally excites Trp and Tyr residues. Taking into consideration that SS bonds are very good quenchers of protein fluorescence, it is likely that their direct or indirect disruption [5–8, 15, 36, 37] leads to an increase of LYZ fluorescence emission intensity when illuminated with wavelengths that lead to direct photolysis of SS (~254 nm) or when illuminated with wavelengths that excite their aromatic residues. The excitation of Trp and Tyr aromatic residues has the highest probability to induce SS disruption since the yield of electron ejection is higher for Trp and Tyr than for Phe residues [6, 36, 37]. Therefore, it is likely that 285 nm and 295 nm excitation of LYZ leads to the largest initial increase of protein fluorescence, followed by 265 nm and 250 nm. It is important to underline “initial increase” since any prolonged excitation of proteins will allow photochemical pathways that can end up bleaching the protein´s fluorescence output. It is likely that 305 nm and, in particular, 310 nm excitation of LYZ does not lead to particular changes of the fluorescence output since these wavelengths lead to a very marginally excitation of LYZ. The data displayed in Fig 6A and 6B corroborates our interpretation.

**Table 7:** Molar extinction coefficients (cm^-1^.M^-1^) at different wavelengths (250 nm, 265 nm, 285 nm, 295 nm, 305 nm and 310 nm) studied for Trp, Tyr and Phe.

	Molar Extinction Coefficients (cm^-1^.M^-1^)		
Wavelength (nm)	Trp	Tyr	Phe
250	2240	274	136
265	4650	921	103
285	4544	675	1
295	1532	61	0
305	150	39	0
310	61	32	0
**Number aromatic residue/ protein**	6	3	3

SYPRO^®^ Orange was selected as a probe in order to monitor LYZ conformational changes, which results in solvent exposure of hydrophobic surfaces [2]. After 70 min of continuous 295 nm excitation of LYZ, SYPRO^®^ Orange fluorescence emission and excitation intensities have increased 25.5-fold (Fig 7A). The increase in fluorescence emission intensity of SYPRO^®^ Orange indicates that this extrinsic probe is in contact with hydrophobic surfaces rendered accessible due to light induced conformational changes in LYZ. Furthermore, continuous excitation leads to a 19 nm blue-shift in the fluorescence emission peak of SYPRO^®^ Orange (Fig 7A). This has also been observed for Nile Red—another hydrophobic fluorescent probe—used to monitor LYZ under stress induced by heat shock (heating at 70°C for 10 min, at pH 5.0), alone and in presence of stabilizers (e.g., betain, hydroxyectoine and trehalose) [2]. The fluorescence emission intensity of SYPRO^®^ Orange at the wavelength where maximum fluorescence emission is observed has been plotted after consecutive 10min illumination periods of LYZ at 295 nm (Fig 7B). After the first and second initial illumination cycles the fluorescence emission intensity of SYPRO^®^ increases by 5.2-fold and 23.5-fold, respectively, indicating that LYZ is in two different conformational states that have in common the fact that hydrophobic surfaces of LYZ became accessible to SYPRO^®^ Orange and, therefore, were solvent accessible. The third cycle of LYZ illumination (30 min) leads to a conformation with a smaller area of hydrophobic surface accessible to the solvent than the area accessible at the end of the second cycle (20 min). After 40 min of illumination the fluorescence emission intensity of SYPRO^®^ Orange at 580 nm increased and stabilized (Fig 7B) indicating that the conformation of LYZ is most likely not to exhibit further changes.

The dependence of the fluorescence emission intensity trace upon continuous 295 nm excitation on excitation power is displayed in Fig 8. At higher power levels (0.7 μW and 1.0 μW) two significant changes in fluorescence emission intensity is observed. Data displayed in Fig 9A clearly show that illumination carried out with the lowest power (0.1 μW) does not lead to significant photochemistry and damage of the Trp pool of residues due to the fact that the fluorescence excitation and emission spectra prior and after 2 hours illumination are very similar. Furthermore, no spectral shift is observed in the fluorescence emission spectrum of LYZ, indicating that the solvent accessibility of the Trp residues in LYZ has not changed (Fig 9A). On the other hand, when illumination is carried out with 1.0 μW power (Fig 9B) it is clear that 295 nm excitation has induced structural changes in LYZ. The intensity of the fluorescence excitation and emission spectra has decreased (36.6% and 11.5%, respectively) after 2hours illumination and the fluorescence emission has been 4 nm red shifted (Fig 9B), indicating that the Trp moiety in LYZ has been rendered more solvent accessible due to light induced protein conformation changes. Wu et al. 2008 [9] reported the photo induced degradation of LYZ (280 nm) as a function of illumination time, at pH 8.0 and 25°C, showing that Trp fluorescence emission intensity increased as a result of continuous excitation, which is correlated with the disruption of SS bridges. A red-shift was also observed. The drop in fluorescence emission intensity is correlated with the formation of Trp photoproducts like NFK and Kyn upon 295 nm excitation of LYZ, as shown in Fig 16A and 16B. Data confirm that the light induced structural damage is dependent on the power of the light used to excite the molecules [8]. Our data indicate that LYZ is not exhibiting photo-oxidation changes at 0.1 μW at 295 nm illumination. However, an increase to 0.5 μW leads to distinct changes.

The study of the effect of temperature (10°C, 20°C, 25°C and 30°C) on LYZ fluorescence emission intensity as a function of 295 nm illumination time, at a fixed slit size (0.5 mm, or 1.0 μW), has been carried out. The reversibility of the processes has also been investigated upon exposing LYZ to repeated 10 min cycles of alternated excitation and darkness. LYZ kinetic traces showed a clear temperature-dependent behaviour. At 20°C the light induced a reversible loss of fluorescence emission intensity. As displayed in Fig 10, after each 10 min dark period LYZ has recovered the fluorescence emission intensity value at the 20 min previous moment where the shutter have been open. This was not observed at any other tested temperature. At 10°C the fluorescence emission intensity increases with illumination time and at 25°C and 30°C the observed increase is minimal.

SYPRO^®^ Orange has been used to monitor the conformational changes of LYZ as a function of temperature. Data show that 2 hours-excitation of LYZ at 295 nm at four different temperatures induces different conformational changes in LYZ (Fig 11). SYPRO^®^ Orange showed highest fluorescence emission intensity at 10°C and lowest fluorescence emission intensity at 20°C. The conformational changes that led to the largest exposure of hydrophobic areas in LYZ has occurred at 10°C, followed by 30°C, 25°C and finally 20°C. At 20°C the lowest exposure of hydrophobic patched in LYZ has occurred. Interestingly, this is the temperature at which the loss of fluorescence emission intensity seems to be reversible upon keeping LYZ in the darkness (Fig 10). In order to confirm such reversible process, we have recorded the fluorescence emission intensity of the following samples: a) fresh LYZ sample, b) fresh sample after being excited for 30 min at 295 nm, c) fresh sample after being excited for 30 min at 295 nm and kept in the dark for 48 hours, d) fresh sample after being excited for 30 min at 295 nm, kept in the dark for 48 hours and further excited for another 30 min with 295 nm. Such experiments took place at 10°C and 20°C. Data displayed in Fig 12A and 12B confirm that the loss of protein fluorescence after the first 30 min of excitation is reversible if the sample is kept in the dark and the experiment is carried out at 20°C and non-reversible if the experiments have been carried out at 10°C.

In the present study we have also immobilized LYZ onto gold nanoparticles coated with hyaluronic acid (HA) and oleic acid (OA). Since gold, HA and OA are known to be fluorescence quenchers, we have investigated if the presence of such quenchers decreased the rate of the observed photochemical reactions and if it induced a preference for short fluorescence decay lifetimes in the case of the conjugated LYZ compared to free LYZ. Data displayed in Figs 13 and 15 show that the presence of gold nanoparticles coated with HAOA (Fig 14A) quenches LYZ fluorescence. The results summarised in Table 5 and the kinetic traces displayed in Fig 13 confirm that the rates of fluorescence emission intensity loss (k1 and k2 values, Table 5) as a function of 295 nm illumination time are one order of magnitude slower for the conjugated LYZ compared to free LYZ. Furthermore, analysis of the fluorescence lifetimes’ distribution of free LYZ compared to LYZ conjugated to HAOA gold nanoparticles (upon pulsed excitation with 280 nm and 295 nm diodes) reveals that upon conjugation, the percentage of molecules that decays with lifetimes in the picosecond range increases, while the percentage of the population that decays with lifetimes in the nanosecond range decreases (see Table 6). When exciting with a 280 nm diode it is observed that for free LYZ, 21% of the population decays with a picosecond lifetime and 79% of the population decays with a nanosecond lifetime. When conjugated to HAOA gold nanoparticles it is observed that 50% of the population decays with a picosecond lifetime and 50% of the population decays with a nanosecond lifetime. When exciting the samples with a 295 nm diode it is observed that for free LYZ, 26% of the population decays with a picosecond lifetime and 74% of the population decays with a nanosecond lifetime. When conjugated to HAOA gold nanoparticles it is observed that >91% of the population decays with a picosecond lifetime and only 9% of the population decays with a nanosecond lifetime. LYZ proximity to the HAOA gold nanoparticles creates a different physico-chemical environment that promotes a faster decay from LYZ excited state. It is known that both silver and gold nanoparticles quench protein fluorescence [16, 38] and that the presence of nanoparticles leads to protein conformational changes revealed by shifts in the fluorescence emission spectrum of the protein [39]. Furthermore, it is known that both HA and OA are good fluorescence quenchers and that their presence leads to shorter fluorescence lifetime components [18, 19]. The protonated forms of carboxylic, hydroxyl and amine groups are known to be fluorescence quenchers of proteins [40]. These groups are present in the glucuronic acid and N-acetyl-glucosamine monomers in the hyaluronic acid (HA) polymer and in oleic acid (OA) and will therefore promote shorter fluorescence lifetime decays in proteins.

Furthermore, as observed in Fig 15, the conjugation of LYZ to HAOA gold nanoparticles leads to protein conformational changes since a blue shift (from 341 nm to 326 nm) in the fluorescence emission spectra is observed upon conjugation. The observed blue indicates that the Trp moiety in LYZ is in a more hydrophobic environment upon conjugation. The hydrophobic environment can be created by the presence of aliphatic chains in OA and by the presence of HA. Such new conformational states might also be responsible for the observed shorter lifetime decays in conjugated LYZ due to a putative closer spatial distance between the aromatic residues and neighboring quencher residues.

In addition, other studies involving binding of drugs (e.g., ciprofloxacin and lomefloxacin) and other molecules (e.g, kynurenine) to LYZ are likely to induce conformational changes on this protein, through formation of aggregates [40] and complexes [39, 41]. These complexes modify the rate of lifetime decay compared to the equivalent components in their non-complex state.

The average fluorescence lifetime (at 330 nm) for free LYZ at pH 6.0 was 1.86 nanosecond and 1.5 nanosecond upon 280 nm and 295 nm excitation, respectively. The fluorescence decay data was best fitted with a 3 exponential decay model. When changing from a 2 lifetime decay model to a 3 lifetime decay model the statistics of the fitting routine improved considerably. For example, the Durbin-Watson parameter improved from being to 1.3 to being 1.8, being a value close to 2 ideal since it shows that the residual are not autocorrelated [42]. Choosing a 4 exponential decay model did not improve the statistics of the fitting routine, except for LYZ-conjugated gold nanoparticles excited at 295 nm. Free LYZ has been previously reported to be best fitted by a three exponential decay model, at a similar pH (5.5) and at 340 nm emission, with an average lifetime of 1.16 nanosecond [43]). Quenching of other proteins besides LYZ by nanocarriers has been reported in literature [16, 44, 45]. In our study both the gold nanoparticles and the coating polymers act as quenchers, contributing to the prevalence of short lifetime components. It has been previously described that the attachment of LYZ to gold nanostructures leads to the appearance of a shorter fluorescence lifetime than the shortest lifetime observed for free LYZ and that the longest fluorescence lifetime becomes longer compared with native LYZ, upon 280 nm excitation and when emission is fixed at 360 nm [44]. Our results are in accordance with this observation (Table 6). This effect can be associated with the quenching effect of gold nanoparticles associated with energy transfer to the gold surface [44].

The quenching effect by HAOA gold nanoparticles on LYZ fluorescence emission intensity (Fig 15), the shorter fluorescence lifetime components observed (Table 6) in the presence of the nanocarriers and the slower kinetics observed on conjugated LYZ upon continuous 295 nm illumination compared to free LYZ (Fig 13 and Table 5) indicates that these particles protect LYZ against photochemistry. It is likely that the structure of LYZ when conjugated to the HAOA gold nanoparticles will be able of being UV excited for longer time prior to possible loss of structure and function. Furthermore, as displayed in Fig 16A and 16B, the amount of photoproducts formed (NFK and Kyn) upon continuous 295 nm excitation of LYZ is reduced in the presence of the nanoparticles.

Previous studies using LYZ mounted on silver nanoparticles also report that quenching can occur as a result of complexation between protein and nanocarrier, as the presence of increasing concentration of silver nanoparticles decreases LYZ fluorescence intensity [38]. LYZ can be linked to gold nanoparticles by non-covalent interactions such as hydrophobic, van der Waals, electrostatic and hydrogen bond interactions [45]. When conjugated to HAOA gold nanoparticles, LYZ will also bind to the HAOA polymer, as represented in Fig 14A. In Fig 14B a TEM image of HAOA gold nanoparticles (without LYZ) is displayed. The gold core (dark core) and the surrounding polymer can be observed. A 3D display of the TEM image shows such two regions in both particles (Fig 14C). Fig 14B and 14C confirm that the gold nanoparticles are covered by a polymer layer. Since LYZ shows a pI around 11.0 and HAOA gold nanoparticles have a superficial negative charge (-19 mV), attractive electrostatic interaction between the protein and the nanocarrier will occur at pH 7.4. Protein conjugation onto the particles has been confirmed by fluorescence spectroscopy (Fig 15). Conjugation has been made possible due to the electrostatic interaction between negatively charged HAOA polymer and positively charged LYZ. The fluorescence excitation (acquired fixing em. at 330 nm) and emission (upon exc. at 295 nm) intensity spectra of free LYZ, of the supernatant recovered after centrifugation of the solution containing conjugated and free LYZ, and of conjugated LYZ onto HAOA gold nanoparticles has been recorded (Fig 15). Data confirm the presence of LYZ onto the HAOA gold nanoparticles. A blue shift of 15 nm in the fluorescence emission spectrum was visible after conjugation of LYZ with HAOA gold nanoparticles. Ali et al. [38] have also described a similar effect (3 nm blue shift) after conjugation of LYZ onto silver nanoparticles. Both temperature and quenchers (i.e., HAOA gold nanoparticles) have an impact on LYZ conformation and structure. When LYZ is in the proximity of HAOA gold nanoparticles, we detect protein conformational changes that reveal that the Trp residues are in a more hydrophobic environment. This environment is provided by the HAOA gold nanoparticles and may increase the interactions and the binding affinity between the Trp moiety and the HAOA gold nanoparticles. This has also been referred by other authors in literature [16]. Concerning the thermal effect on LYZ, Trp residues become more solvent accessible, indicating that the hydrophobic core is more exposed to the solvent, which may increase the formation of inter-protein interactions [46].

Charged amine groups seem to play an important role in the conjugation, since they will interact with negatively charged acid groups present in the used polymer, hyaluronic acid, and present in oleic acid at pH 7.4 [20, 47]. The use of natural polymers has an important and advantageous role in the reduction and morphology of gold nanoparticles (e.g., can work as capping agents, activate “green” reduction of gold and are less toxic), as reported in literature [47]. Conjugation of LYZ onto gold nanorods for 2 hours at pH 6.2 and room temperature led to a blue shift in the nanorods absorbance spectrum and the nanorods acted as quenchers of Trp fluorescence [16]. When monitored at 35°C and pH 7.25, LYZ activity increased when conjugated with iron oxide superparamagnetic nanoparticles, which was observed to be correlated with an increase in the amount of β-sheets and α-helix coils [48]. Another study with iron oxide superparamagnetic nanoparticles demonstrated no change in aromatic residues of LYZ and protein activity after adsorption at the nanocarriers surface by incubation at 37°C for 60 min (200 rpm) [49].

The presence of oxidative conditions induced by light and by the presence of metallic surfaces can lead to the oxidation of the aromatic residues in proteins [5–7, 36, 50–52]. UVB excitation of aromatic residues in proteins leads to the disruption of SS bridges [5, 7, –8, 14, 36,37] and to the formation of photoproducts, such as NFK, Kyn [25, 53] and DT [26]. The spectral properties of these species are displayed in Table 1. In Fig 16A and 16B are displayed the fluorescence emission spectra obtained upon 320 nm and 360 nm excitation, respectively, of a fresh LYZ sample and of a LYZ-HAOA gold nanoparticles sample before and after 2 hours of 295 nm excitation. Since 295 nm excites specifically Trp residues, it is most likely that the photoproducts formed are Trp derivatives such as NFK and Kyn and not Tyr derivatives like DT. Furthermore, the emission spectrum of LYZ upon 295 nm leads to a fluorescence emission spectrum that peaks around 340 nm with a very small component below 290 nm. Therefore, it is unlikely that Tyr residues will be excited by LYZ emission. Two excitation wavelengths were used: 320 nm (Fig 16A) and 360 nm (Fig 16B). Light at 320 nm excites both NFK (ε_NFK(321nm)_ = 3750 M^-1^cm^-1^ [54–57] and Kyn (ε_Kyn(321nm)_ = 1812 M^-1^cm^-1^ [58]. At 315 nm DT has an extinction coefficient equal to 5200 M^-1^cm^-1^, but as explained above it is unlikely that we have DT formation [59, 60]. Light at 360 nm excites NFK (ε_NFK(360nm)_ = 1607 M^-1^cm^-1^ [58] and Kyn (ε_Kyn(365nm)_ = 4530 M^-1^cm^-1^ [61, 62]) but does not excite DT. Therefore, the peak with maximum fluorescence emission intensity at 405 nm in Fig 16B cannot be due to the presence of DT, since DT is not excited at 360 nm. This peak has to belong to a Trp photoproduct that can absorb light both at 320 nm and at 360 nm, since the peak is presence both in Fig 16A and 16B. It cannot be Kyn since the wavelength of maximum fluorescence emission of Kyn lies within 434–480 nm. According to Fukunaga et al., Kyn62LYZ (where Trp62 was converted into Kyn62 in lysozyme, [25]) fluoresces maximally at 470 nm and has broad fluorescence emission spectrum upon excitation at 360 nm. Thus, results are in accordance with ours since we see a broad fluorescence emission centered approximately at 466 nm (Fig 16B). Furthermore, it is reported that Kyn is quenched by protonated amino groups, leading to poor Kyn fluorescence [25]. The 466 nm broad peak in Fig 16B indeed displays poor fluorescence and LYZ at pH 7.4 has protonated amino groups (ARG61 and ARG112) that can quench Kyn. The closest distances between ARG61 (NE) and ARG112 (NE) to the nearby TRP62 (CZ2) and TRP111 (CZ3) are 4.3 Å and 7.5 Å, respectively. Our quest is to find the origin of the 406 nm peak observed in Fig 16A and 16B. NFK absorbs light both at 320 nm and 360 nm and NFK emission is reported to be more blue shifted (maximum emission between 400–440 nm) than Kyn (maximum emission between 434–480 nm). The NFK emission peak is very dependent on pH [25], and on the local dielectric constant (low dielectric medium leads to a blue shift of the maximum emission wavelength, [25]). When exposed to an alkaline pH, NFK emits maximally at 400 nm and when exposed to a neutral pH it emits maximally at 440 nm [25]. On the other hand, NFK emission is not quenched by protonated amino groups which lead to an enhancement of NFK fluorescence. The peak at 406 nm has indeed a large fluorescence emission intensity compared to the Kyn peak centered at 466 nm upon 360 nm excitation (Fig 16B). Summarizing, in Fig 16A and 16B it is likely that the peak at 405–406 nm is due to the formation of NFK upon 295 nm continuous excitation of both free LYZ (Fig 16A and 16B, blue spectra) and conjugated LYZ (Fig 16B, green spectra). According with the results of Fukunaga et al. [25], the broad peak with maximum fluorescence at 433 nm seen in Fig 16A for conjugated LYZ (green curves) is due to the presence of NFK.

In Fig 16A and 16B it can also be seen that the amount of photoproducts formed upon 295 nm excitation of LYZ is reduced when LYZ has been conjugated to HAOA gold nanoparticles. It again likely that the additional presence of fluorescence quenchers such as gold, HA and OA by reducing the fluorescence lifetime of LYZ, prevents that excited Trp residues enter photochemical pathways that lead to the formation of e.g. NFK and Kyn. As a result of LYZ close vicinity to fluorescence quenchers it is likely that static quenching occurs due to the formation of complexes between LYZ and HAOA gold nanoparticles [39, 40]. Plasmonic surfaces such as the gold and biomolecules such as HA and OA are described to be good fluorescence quenchers [16, 18, 19]. In our studies, the occurrence of static quenching between LYZ and HAOA gold nanoparticles leads to the shorter fluorescence lifetimes for conjugated LYZ (Table 6), to the reduction of LYZ fluorescence intensity, to a fluorescence emission blue shift from 341 nm to 326 nm (Fig 15) after 2 hours continuous illumination at 295 nm and, finally, to the prevention of excited Trp residues to enter photochemical pathways that lead to the formation of NFK and Kyn, in the case of LYZ-conjugated HAOA gold nanoparticles (Fig 16A and 16B).

## Conclusions

With this work, it has been demonstrated that the photochemical effects occurring during 295 nm UV excitation at 20°C are reversible, but not at 10°C, 25°C and 30°C. This paper also provides evidence that the UV-damage threshold is between 0.1 μW and 0.5 μW for LYZ. In addition, we have developed HAOA gold nanoparticles that can efficiently protect proteins like LYZ from 295 nm induced photochemistry. These results can be used for biomedical application, as gold nanoparticles gain a greater impact as drug delivery platforms, in areas such as targeting delivery and use of peptides and biomolecules as specific ligands for target cell pools or simply as enzyme carriers. Further, we will study the application of other peptides conjugated with the developed HAOA gold nanoparticles, as biologic triggers for application in cancer laser-based photothermal treatment.
